# Loss of miR-145 promotes remyelination and functional recovery in a model of chronic central demyelination

**DOI:** 10.1038/s42003-024-06513-x

**Published:** 2024-07-04

**Authors:** Samantha F. Kornfeld, Sarah E. Cummings, Rebecca Yaworski, Yves De Repentigny, Sabrina Gagnon, Stephanie Zandee, Samaneh Fathi, Alexandre Prat, Rashmi Kothary

**Affiliations:** 1 Regenerative Medicine Program, Ottawa Hospital Research Institute, Ottawa, ON K1H 8L6 Canada; 2 Department of Cellular and Molecular Medicine, University of Ottawa, Ottawa, ON K1H 8M5 Canada; 3 Neuroimmunology Unit and Multiple Sclerosis Clinic, The Research Center of the Centre Hospitalier de l’Université de Montréal (CRCHUM), Department of Neuroscience, Faculty of Medicine, Université de Montréal, Montréal, QC Canada; 4 Department of Medicine, University of Ottawa, Ottawa, ON K1H 8M5 Canada; 5 Centre for Neuromuscular Disease, University of Ottawa, Ottawa, ON K1H 8M5 Canada

**Keywords:** Multiple sclerosis, Gene regulation

## Abstract

Strategies for treating progressive multiple sclerosis (MS) remain limited. Here, we found that miR-145-5p is overabundant uniquely in chronic lesion tissues from secondary progressive MS patients. We induced both acute and chronic demyelination in miR-145 knockout mice to determine its contributions to remyelination failure. Following acute demyelination, no advantage to miR-145 loss could be detected. However, after chronic demyelination, animals with miR-145 loss demonstrated increased remyelination and functional recovery, coincident with altered presence of astrocytes and microglia within the corpus callosum relative to wild-type animals. This improved response in miR-145 knockout animals coincided with a pathological upregulation of miR-145-5p in wild-type animals with chronic cuprizone exposure, paralleling human chronic lesions. Furthermore, miR-145 overexpression specifically in oligodendrocytes (OLs) severely stunted differentiation and negatively impacted survival. RNAseq analysis showed altered transcriptome in these cells with downregulated major pathways involved in myelination. Our data suggest that pathological accumulation of miR-145-5p is a distinctive feature of chronic demyelination and is strongly implicated in the failure of remyelination, possibly due to the inhibition of OL differentiation together with alterations in other glial cells. This is mirrored in chronic MS lesions, and thus miR-145-5p serves as a potential relevant therapeutic target in progressive forms of MS.

## Abstract (web summary)

Loss of miR-145 promotes remyelination in a chronically demyelinated animal model, implicating it as a factor in remyelination failure in human Multiple Sclerosis lesions and highlighting in as a potential therapeutic target for progressive MS.

## Introduction

Multiple sclerosis (MS) is a progressive demyelinating disease of the central nervous system (CNS) affecting approximately 2.5 million people worldwide. Understanding of the aetiology of MS remains incomplete, but its pathophysiology is underpinned by aberrant CNS entry of auto-reactive T- and B-cells in genetically pre-disposed individuals, which may be triggered by one or more environmental factors such as vitamin D deficiency, viral infection, childhood obesity, and/or smoking^1^. Neurological damage accumulates over time and results in both physical and cognitive deficits. In the more common relapsing-remitting MS (RRMS), symptoms are largely experienced during a period of autoimmune attack that spurs a cascade of neuroinflammation, followed by full or partial functional recovery^1–3^. During the disease course, this cycle of relapse and remission will transition to secondary progressive MS (SPMS) for the majority of patients, at which point remissions cease to occur and accumulated disabilities become permanent^2^. A subset of patients presents with primary progressive MS (PPMS) at disease onset; symptoms in these patients simply worsen over time with no periods of remission^2,3^.

Current first-line treatment options available to MS patients include immunomodulatory therapies aimed at reducing CNS inflammation to limit myelin damage^4,5^. However, these treatments show varying efficacy, and largely only in RRMS patients—treatment for progressive MS patients remains discouragingly limited. Further, the fact that disease continues to progress in RRMS patients even with treatment indicates that alternative strategies for both RRMS and progressive MS require rigorous ongoing research. Advances have been made in both hematopoietic and mesenchymal stem cell transplants for MS treatment, offering riskier but potentially long-term resolution from myelin attack^6–8^. However, these are so far only indicated for patients for whom first-line therapies prove ineffective and are experiencing active inflammatory disease - as is seen most often in RRMS - again, not improving therapeutic options for progressive MS patients^7^.

Though the resultant symptoms of MS autoimmune attack are attributable to a loss of neuronal signalling efficiency and/or neurodegeneration, these are secondary to loss of myelin and the CNS producers of myelin, oligodendrocytes (OLs). In a healthy individual and in a person with RRMS, a demyelinating event is followed by the recruitment of oligodendrocyte progenitor cells (OPCs) distributed throughout the CNS to the demyelinated area; these cells then differentiate into OLs and remyelinate denuded axons^9–11^. Thus, in RRMS, periods of remission are in fact periods of remyelination. Conversely, in progressive MS, remyelination is limited to non-existent. This leaves neurons unprotected and without trophic support, leading to eventual neurodegeneration and irreversible disability^12^. Consequently, remyelination strategies in demyelinating diseases such as MS remain an important opportunity for the expansion of treatment options. Such therapies could benefit patients with RRMS through a combination of therapeutics to both relieve immune-mediated attack on myelin and enhance remyelination, and progressive MS patients for whom there are currently no effective medications to slow disease progression.

Reasons for remyelination failure are multi-faceted. Recruitment of OPCs to the lesion area may in some instances be impaired due to a loss of chemoattractants such as Semaphorin A^13^. Most often though, lesions in chronic and progressive disease are replete with OPCs and incompletely differentiated OLs, indicating that a lack of remyelination-capable cells is not the cause for lack of remyelination^14–16^. It appears instead that the terminal differentiation of these OPCs and early OLs is inhibited due to the inhospitable microenvironment of the lesion; factors such as chondroitin sulphate proteoglycans (CSPGs), basic fibroblast growth factor (FGF2), inflammatory cytokines and myelin debris present at high levels in lesion tissue all negatively regulate OL differentiation^17–20^.

Previously, we characterized miR-145-5p as a negative regulator of OL differentiation in primary OLs in vitro, where its expression is required to maintain OPCs in their proliferative and undifferentiated state and its downregulation promotes enhanced OL differentiation^21^. Based on unvalidated microarray data from Junker et al., miR-145-5p is overabundant in chronic inactive lesions from progressive MS patients relative to both healthy controls and active lesions from RRMS patients, suggesting it may be a discriminating difference between the remyelination-permissive versus non-permissive environments of RRMS tissues and progressive MS tissues, respectively^22^. Taken together, we hypothesized that miR-145-5p may be a defining factor of myelination failure, and that loss of miR-145-5p expression might promote remyelination in vivo. Thus, with the current study, we sought to validate the expression of miR-145-5p in progressive MS lesion tissue and determine whether its loss affects the course of remyelination using both acute and chronic pre-clinical models of demyelination.

## Results

### miR-145-5p levels are elevated in chronic inactive MS lesion tissue

**Fig. 1:**
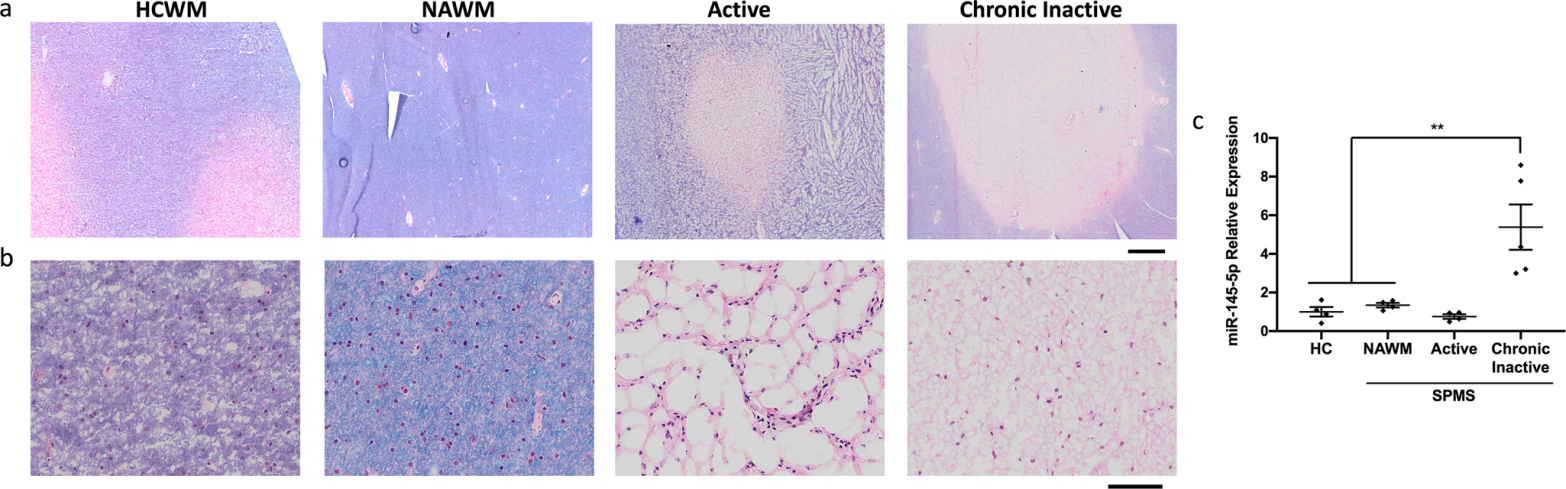
miR-145-5p is upregulated in chronic lesion tissue from SPMS brains. **a**, **b** Luxol fast blue/hematoxylin and eosin stained human brain tissue from healthy control white matter (HCWM), and normal appearing white matter (NAWM), active and chronic inactive lesion tissue from SPMS brain. **a** Scale bar = 1 mm. **b** Magnified from (**a**); scale bar = 50 µm. **c** Relative expression of miR-145-5p in HC and NAWM, active and chronic inactive lesion tissue from SPMS brain. Analysed by ΔΔCt, normalized to snU6. *N* = 4–5, **p < 0.01, one-way ANOVA with Tukey’s *post hoc*.

Comparison of transcripts present in acute or chronic MS lesions identified miR-145-5p as an overabundant factor in chronic lesions^22^. Given that we have previously shown that miR-145-5p expression inhibits OPC differentiation to OLs, we reasoned that increased levels of miR-145-5p might contribute to remyelination failure in chronic lesions. To further assess this possibility, we performed expression analysis for miR-145-5p in tissues from 4 healthy controls (HCs), 4 SPMS patients and 1 RRMS patient (Table 1). Tissues assessed from SPMS and RRMS included normal appearing white matter (NAWM), active lesion tissue and chronic inactive lesion tissue (Fig. 1a, b). All chronic inactive lesions demonstrated an increase of miR-145-5p ranging from 3.00- to 8.59-fold change relative to HC, while NAWM and active lesions exhibited miR-145-5p expression similar to HC (Table 1; Fig. 1c). Despite this small patient sample set, we confirm from this experiment that miR-145 is significantly elevated in chronic inactive lesions suggesting that it can contribute to remyelination failure and MS progression.

**Table 1:** Expression of miR-145-5p is elevated in chronic inactive lesion tissue from SPMS and RRMS patients

Disease	Sex	Age (yrs)	Disease duration (yrs)	EDSS^a^	Tissue type	miR-145-5p expression ± SEM	FC^b^/mean HC^c^
SPMS^d^	M	61	26	9.5	NAWM^f^	0.67 ± 0.01	1.58
					Chronic Inactive	1.27 ± 0.03	3.00
					Active	0.39 ± 0.04	0.93
SPMS	M	65	13-15	9	NAWM	0.45 ± 0.01	1.06
					Chronic Inactive	3.64 ± 0.20	8.59
					Chronic Inactive	3.29 ± 0.12	7.77
					Active	0.20 ± 0.01	0.48
SPMS	F	60	28	8.5	NAWM	0.53 ± 0.01	1.25
					Chronic Inactive	1.36 ± 0.08	3.21
					Active	0.41 ± 0.02	0.96
SPMS	M	48	6	8.5	NAWM	0.63 ± 0.03	1.48
					Chronic Inactive	1.84 ± 0.05	4.36
					Active	0.27 ± 0.01	0.63
RRMS^e^	M	26	12	9	NAWM	0.43 ± 0.01	1.03
					Chronic Inactive	2.28 ± 0.03	5.40
					Active	0.33 ± 0.02	0.79
HC	M	64	--	--	WM^g^	0.17 ± 0.00	--
HC	M	67	--	--	WM	0.46 ± 0.02	--
HC	F	37	--	--	WM	0.68 ± 0.02	--
HC	M	42	--	--	WM	0.38 ± 0.01	--

^a^Expanded Disability Status Scale.

^b^Fold Change.

^c^Healthy Control.

^d^Secondary Progressive Multiple Sclerosis.

^e^Relapsing-Remitting Multiple Sclerosis.

^f^Normal Appearing White Matter.

^g^White Matter.

### Loss of miR-145 accelerates developmental OL differentiation in vitro but not in vivo

To assess the requirement for miR-145 in OL maturation and CNS myelination we obtained *miR-145*^*–/–*^ animals for analysis. First, we confirmed the loss of miR145a (the mouse ortholog of miR-145; henceforth referred to as miR-145) in CNS tissues by qRT-PCR in *miR-145*^*–/–*^ animals, with the expected Mendelian expression of ~50% in *miR-145*^*+/–*^ animals relative to *miR-145*^*+/+*^ (Supplementary Fig. 1a). We additionally confirmed that miR-145b, a putative second miR-145 gene predicted only in mice, was not detectable in this model (Supplementary Fig. 1a). *MiR-145*^*–/–*^ mice do not exhibit any overt phenotypes. Survival up to one year is normal, as are total body and brain growth during development (Supplementary Fig. 1b–d).

**Fig. 2:**
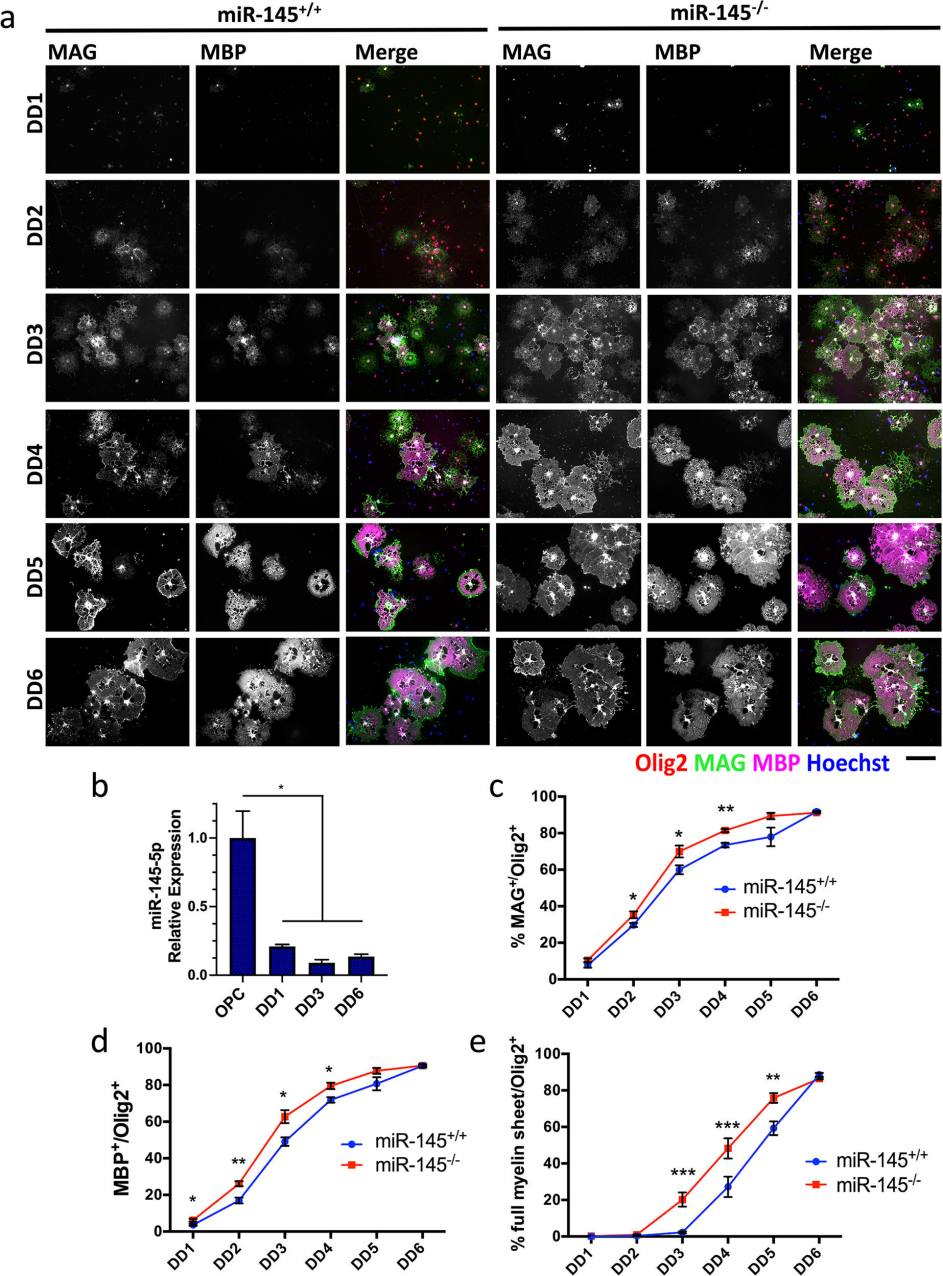
Loss of miR-145 accelerates OL differentiation in vitro. **a** Fluorescence micrographs of primary miR-145^+/+^ (left panels) and miR-145^–/–^ (right panels) primary mouse oligodendrocytes (OLs) at differentiation day 1 (DD1), DD2, DD3, DD4 DD5 and DD6. Cells labelled for Olig2 (red), MAG (green in merge), MBP (magenta in merge) and counterstained with Hoechst. Scale bar = 100 μm. **b** Relative expression of miR-145-5p in Oli-Neu immortalized mouse oligodendrocyte progenitor cells (OPCs) and OLs at DD1, DD3 and DD6. Analysed by ΔΔCt, normalized to *snU6*. *N* = 4, *p < 0.05, one-way ANOVA with Tukey’s *post hoc*. Quantifications of % MAG^+^/Olig2^+^ (**c**), % MBP^+^/Olig2^+^ (**d**) and % full myelin sheet/Olig2^+^ cells at DD1-DD6 (**e**). *N* = 3–4, *p < 0.05, **p < 0.01, ***p < 0.001, multiple t-tests of two-way ANOVA using Holm-Sidak method. All values represent mean ± SEM.

To observe the effects of miR-145 loss selectively during OL differentiation, primary OPCs were isolated from *miR-145*^*+/+*^ and *miR-145*^*–/–*^ neonates and cultured over a time course up to differentiation day 6 (DD6) (Fig. 2a). Differential expression of miR-145-5p was validated in immortalized *Oli-neu* mouse OPCs and DD1, DD3 and DD6 OLs, revealing the expected strong downregulation upon initiation of the differentiation program (Fig. 2b). Myelin marker expression as well as mature morphology were assessed on DD1-6 every 24 h in maturing primary OLs from *miR-145*^*+/+*^ and *miR-145*^*–/–*^ OLs. The proportion of MAG^+^ OLs was significantly increased on DD2-4 and of MBP^+^ OLs was increased on DD1-4 in *miR-145*^*–/–*^ cultures (Fig. 2c, d). By DD5 and DD6 maximal maturity rates were achieved and *miR-145*^*+/+*^ OLs caught up to their knockout counterparts. Similarly, OLs achieving formation of full compact membrane sheets were more numerous in *miR-145*^*−/−*^ OLs on DD3-DD5, with *miR-145*^*+/+*^ OLs again matching *miR-145*^*−/−*^ by DD6 (Fig. 2e).

To determine if miR-145 loss impacted myelin generation in vivo, we measured myelin protein expression in CNS tissues from neonates (postnatal day 0; P0), juveniles (P9 and P15), early adults (P30) and mature adults (P60). CNS tissues were subdivided into forebrain and midbrain (FB/MB), cerebellum and brainstem (C/BS) and spinal cord (SC) to assess their myelin content separately. Immunoblotting was used to assess myelin proteins associated with intermediate myelin formation (CNP + ), late myelin formation (MBP + ), and final internode assembly (MOG + ) at the aforementioned timepoints. At P0, only CNP was detectable, and no differences were found between *miR-145*^*+/+*^ and *miR-145*^*−/−*^ in any CNS tissues (Supplementary Fig. 2). By P9 and onward, MBP and MOG were also expressed at measurable levels; however, no differences were observed at P9, P15, P30 or P60 in any myelin markers assessed in any tissue (Supplementary Fig. 2).

**Fig. 3:**
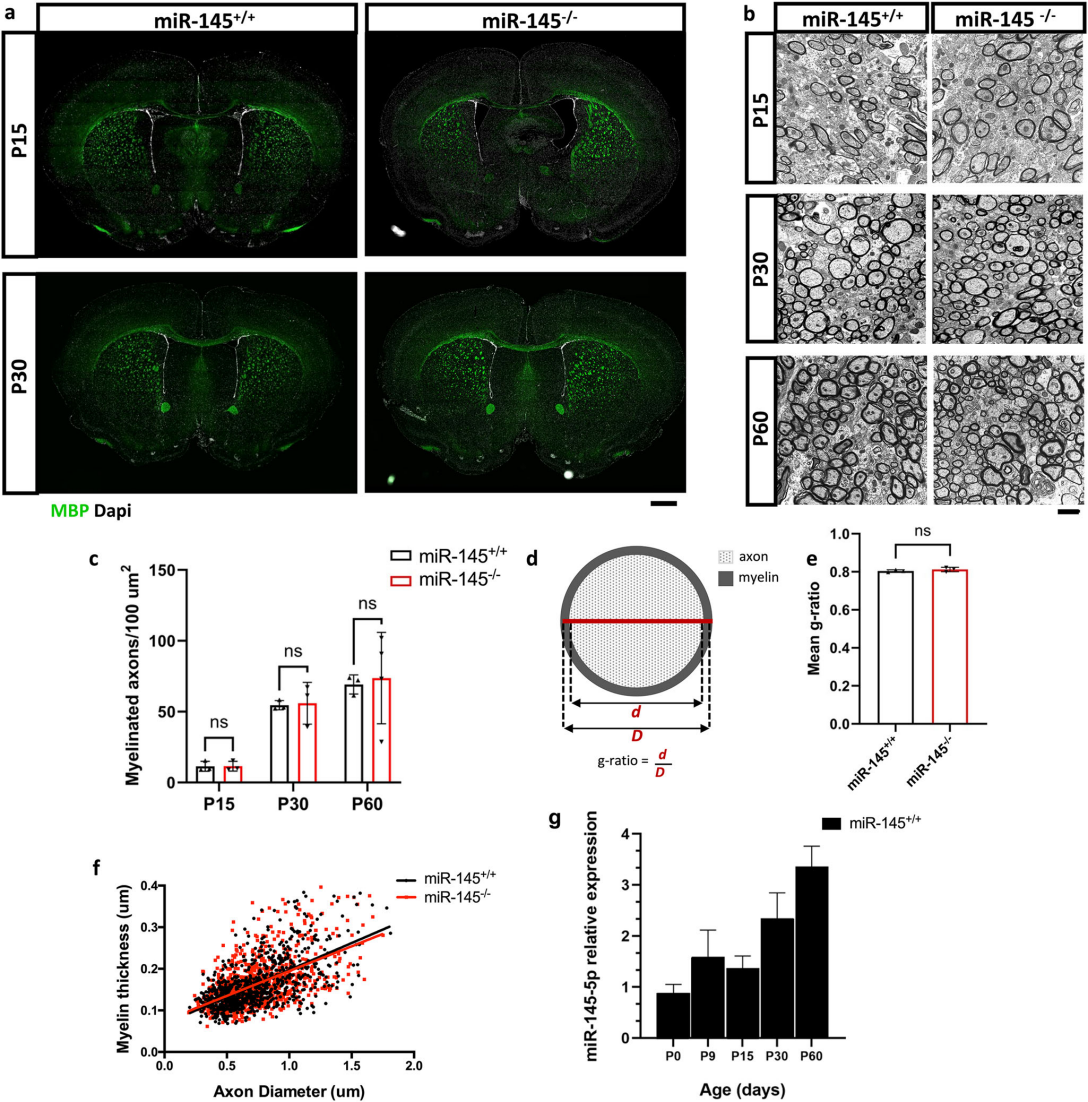
Ultrastructure and number of myelinated axons in corpus callosum is unchanged by loss of miR-145 during development. **a** Fluorescence micrographs of coronal brain sections from juvenile (P15) and early adult (P30) *miR-145*^*+/+*^ and *miR-145*^*–/–*^ animals. Sections stained for MBP (green), counterstained with Dapi (white). Scale bar = 500 μm. **b** TEM micrographs of sagittal sectioned corpus callosum from *miR-145*^*+/+*^ and *miR-145*^*–/–*^ P15, P30 and P60 animals. Scale bar = 2 μm. **c** Quantifications of the numbers of myelinated axons per 100 μm^2^ in corpus callosum from P15, P30 and P60 *miR-145*^*+/+*^ and *miR-145*^*–/–*^ animals. **d** Schematic of g-ratio measurements and calculation. **e** Mean g-ratios of myelinated axons at P60. **f** Linear regression analyses of myelin thickness at P60. **c**, **e** *N* = 3–4, ns not significant by Student’s *t* test, values represent means ± SEM. **g** Relative expression of miR-145-5p in forebrain/midbrain tissues from *miR-145*^*+/+*^ animals at P0, P9, P15, P30, and P60. Analysed by ΔΔCt, normalized to *snU6*. *N* = 3.

To confirm that myelin protein production resulted in functional myelination, we also assessed morphology and myelination of corpus callosum as a hallmark of major white matter structure in juvenile, early and mature adult animals; however, no differences were observed between *miR-145*^*+/+*^ and *miR-145*^*–/–*^ at P15 or P30 (Fig. 3a). Corpus callosum myelination was investigated with greater depth by transmission electron microscopy (TEM) at P15, P30 and P60. No differences could be observed in myelination, and the number of myelinated axons were similar at all time points (Fig. 3b, c, Supplementary Data 1). Finally, g-ratio measurements—calculated as (axon diameter)/(axon + myelin diameter) (Fig. 3d)—from corpus callosum at P60 were not different between *miR-145*^*+/+*^ and *miR-145*^*–/–*^ (Fig. 3e, f). Differential expression of miR-145-5p in forebrain and midbrain tissues was investigated over the course of development from P0 to P60 in *miR-145*^*+/+*^ animals, demonstrating a relatively lower expression level early postnatally at P0 when developmental myelination is occurring at the greatest rate, and increasing gradually over time to ~4-fold by P60 when the bulk of CNS myelin has been established (Fig. 3g). Collectively, these studies indicate that *miR-145*^*–/–*^ mice have no observable defects associated with developmental myelination and that miR-145-5p expression increases over the course of brain development in wild-type animals.

### Loss of miR-145 promotes remyelination and functional recovery following chronic, but not acute, toxic demyelination

To determine whether loss of miR-145 might impact remyelination, we subjected *miR-145*^*+/+*^ and *miR-145*^*–/–*^ animals to both acute and chronic demyelination protocols driven by the copper-chelating agent cuprizone. Cuprizone exposure leads to death of mature OLs and subsequent demyelination primarily in the corpus callosum as well as in adjacent structures such as the cortices and hippocampus. With acute exposure for 6 weeks followed by cuprizone withdrawal, remyelination occurs efficiently, with substantial remyelination occurring 5-6 weeks after withdrawal from cuprizone treatment. In contrast, chronic exposure for 12 weeks typically results in little to no remyelination after cuprizone withdrawal^23,24^.

**Fig. 4:**
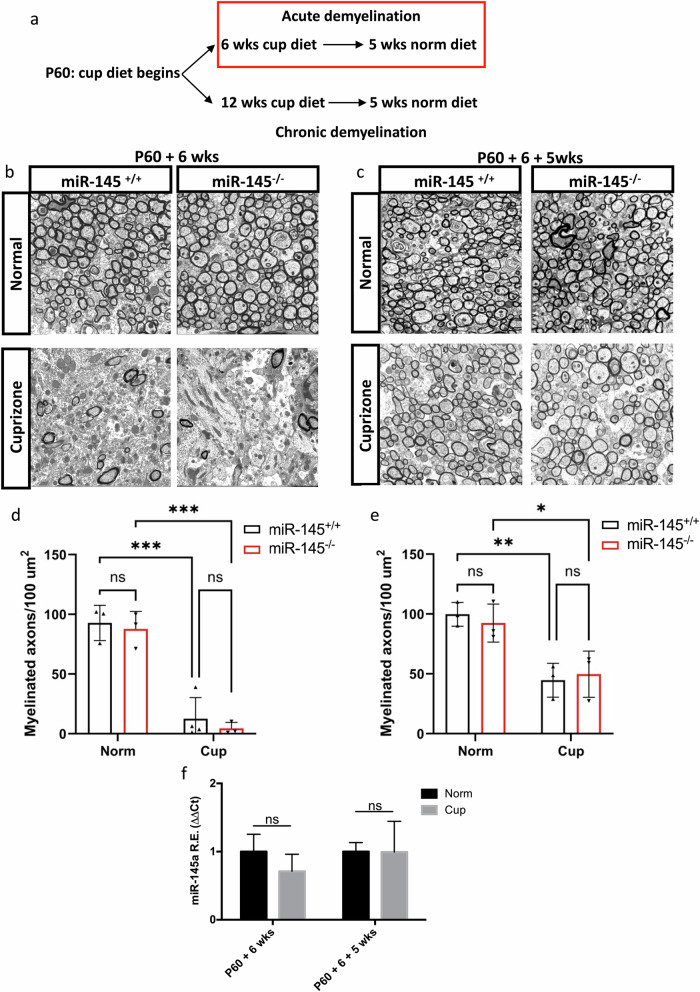
Loss of miR-145 does not affect remyelination following acute cuprizone-mediated demyelination. **a** Schematic of 0.2% cuprizone (cup) treatment timeline and return to normal (norm) diet. TEM micrographs of sagittal sections of corpus callosum from *miR-145*^*+/+*^ and *miR-145*^*–/–*^ animals after 6 weeks (P60 + 6 wks; **b**) on normal diet (Normal) or cuprizone diet (Cuprizone) and after 6 weeks +5 weeks (P60 + 6 + 5 wks; **c**) normal diet (Normal) or 6 weeks cuprizone diet +5 weeks normal diet (Cuprizone). Scale bar = 2 µm. **d**, **e** Quantifications of the numbers of myelinated axons per 100 µm^2^ in normal and cuprizone corpus callosum at P60 + 6 wks (**d**) and P60 + 6 + 5 wks (**e**). *N* = 3–4, two-way ANOVA with Tukey’s *post hoc*. **d**, **e** *p < 0.05, ***p < 0.001. **f** Relative expression (RE) analysis of miR-145-5p in forebrain/midbrain tissue from *miR-145*^*+/+*^ animals after 6 wks cuprizone exposure and after 5 wks return to normal diet. Analysed by ΔΔCt method, normalized to *snU6*. *N* = 3, ns = not significant, Student’s *t* test adjusted for multiple propagations. All values represent mean ± SEM.

We first evaluated myelination in the corpus callosum following 6 weeks cuprizone exposure followed by cuprizone withdrawal and 5 weeks of recovery on a normal diet (Fig. 4a). Demyelination at 6 weeks was extensive in both *miR-145*^*+/+*^ and *miR-145*^*–/–*^ animals, with significant loss of ~90% of myelinated axon numbers (*miR-145*^*+/+*^ 12.6 ± 8.9/100 µm^2^, *miR-145*^*–/–*^ 4.5 ± 2.9/100 µm^2^; Fig. 4 b, d, Supplementary Data 1). After 5 weeks of recovery, remyelination was apparent in corpus callosum from both *miR-145*^*+/+*^ and *miR-145*^*–/–*^ animals with return to ~50% the number of myelinated axons relative to age-matched controls, with no detectable difference between genotypes (*miR-145*^*+/+*^ 44.7 ± 8.1/100 µm^2^, *miR-145*^*–/–*^ 49.7 ± 11.2/100 µm^2^; Fig. 4c, e, Supplementary Data 1). Relative expression of miR-145-5p was similar in normal and acutely demyelinated *miR-145*^*+/+*^ brains both at 6 weeks of cuprizone exposure and after 5 weeks recovery (Fig. 4f).

**Fig. 5:**
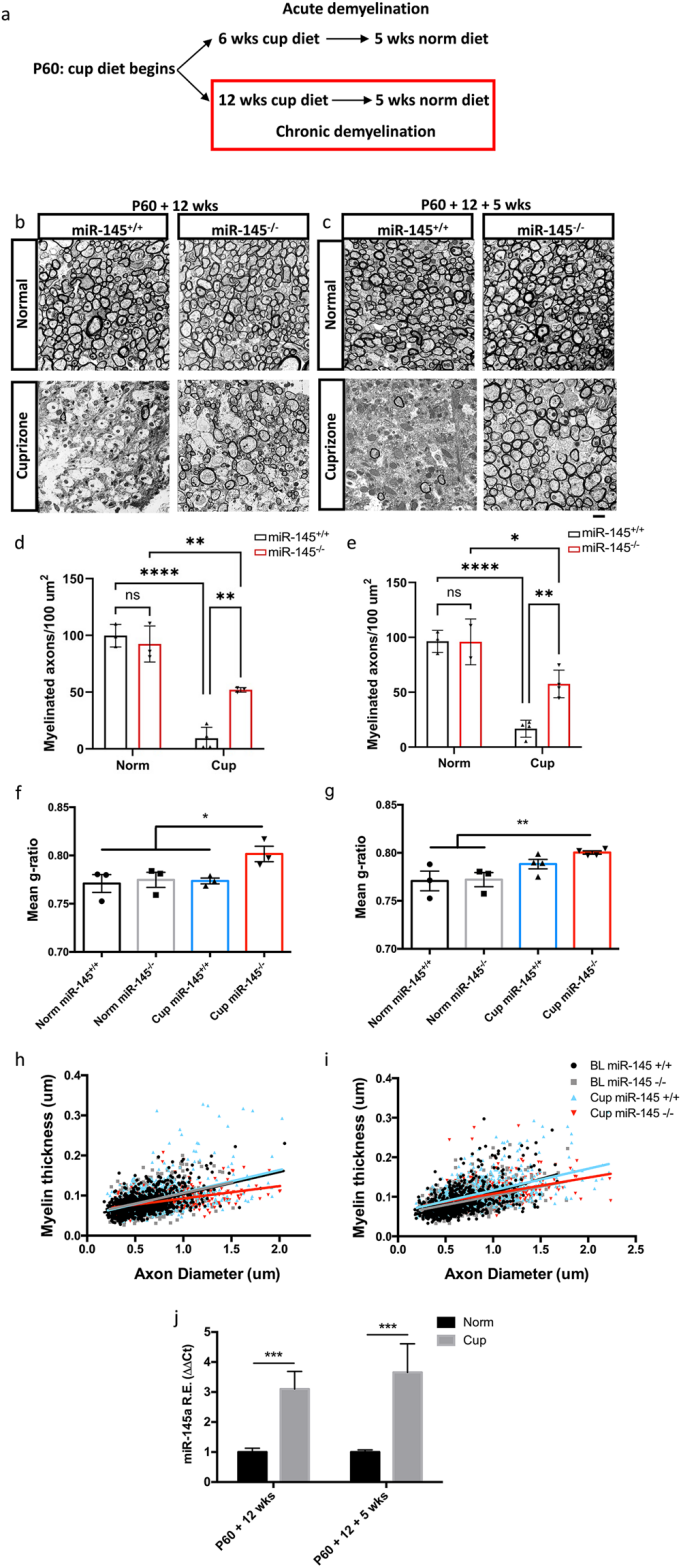
Loss of miR-145 promotes remyelination with chronic cuprizone exposure. **a** Schematic of 0.2% cuprizone (cup) treatment timeline and return to normal (norm) diet. TEM micrographs of sagittal sections of corpus callosum from *miR-145*^*+/+*^ and *miR-145*^*–/–*^ animals after 12 weeks (P60 + 12 wks; **b**) on normal diet (Normal) or cuprizone diet (Cuprizone) and after 12 weeks + 5 weeks (P60 + 12 + 5 wks; **c**) normal diet (Normal) or 12 weeks cuprizone diet + 5 weeks normal diet (Cuprizone). Scale bar = 2 µm. Quantifications of the numbers of myelinated axons per 100 µm^2^ in normal and cuprizone corpus callosum at P60 + 12 wks (**d**) and P60 + 12 + 5 wks (**e**). *N* = 3–4, two-way ANOVA with Tukey’s *post hoc*. Mean g-ratios of myelinated axons at 12 wks (**f**) and 12 + 5 wks (**g**). *N* = 3–4, one-way ANOVA with Tukey’s *post hoc*. **d**–**g** *p < 0.05, **p < 0.01, ***p < 0.001, ****p = <0.0001. Values represent mean ± SEM. Linear regression analyses of myelin thickness at P60 + 12 wks (**h**) and P60 + 12 + 5 wks (**i**) plotted by axon diameter. Legend at i also applies to (**h**). **j** Relative expression analysis of miR-145-5p in forebrain/midbrain tissue from *miR-145*^*+/+*^ animals after 12 wks cuprizone exposure and after 5 wks return to normal diet. Analysed by ΔΔCt method, normalized to *snU6*. *N* = 3, ***p < 0.001, Student’s *t* test adjusted for multiple propagations. All values represent mean ± SEM.

However, following chronic cuprizone exposure we observed significant recovery in the *miR-145*^–*/–*^ animals compared to control. Similar to the acute experiment, after 12 weeks cuprizone exposure (Fig. 5a), *miR-145*^*+/+*^ animals demonstrated ~90% loss of myelinated axons (9.3 ± 4.9/100 µm^2^) relative to healthy age-matched controls (Fig. 5b, d, Supplementary Fig. 3, Supplementary Data 1). By contrast, corpus callosum from *miR-145*^*−/−*^ animals were replete with myelinated axons showing ~50% the number found in controls after 12 weeks cuprizone exposure and ~60% of controls after an additional 5 weeks recovery, while *miR-145*^*+/+*^ cuprizone-treated animals remained sparsely myelinated (P60 + 12 weeks: *miR-145*^*+/+*^ 9.3 ± 4.9/100 µm^2^, *miR-145*^*–/–*^ 52.1 ± 1.2/100 µm^2^; Fig. 5b, d, Supplementary Fig. 3, Supplementary Data 1; P60 + 12 weeks + 5 weeks: *miR-145*^*+/+*^ 16.8 ± 3.9/100 µm^2^, *miR-145*^*–/–*^ 57.6 ± 6.3/100 µm^2^; Fig. 5c, e, Supplementary Fig. 4, Supplementary Data 1). These data indicate that while *miR-145*^*+/+*^ animals became refractory to remyelination with chronic cuprizone exposure, this was not the case for *miR-145*^*–/–*^ animals, irrespective of recovery time.

Myelin produced as a result of remyelination is canonically thinner than developmentally-derived myelin. To confirm this, we measured g-ratios at both 12 weeks cuprizone (Fig. 5f) and 5 weeks recovery (Fig. 5g). At both time points, *miR-145*^–*/*–^ g-ratios were significantly increased relative to control with values indicative of a thinner myelin sheath, calculated at 0.80 ± 0.008 at 12 weeks and 0.80 ± 0.001 after 5 weeks recovery compared to controls which remained steady at ~0.77 (Fig. 5f, g). This is further demonstrated by alterations in the linear regression analyses when plotting myelin thickness by axon diameter from *miR-145*^–*/*–^ in comparison to healthy controls, particularly in larger axons, which typically are preferentially the first to be remyelinated (Fig. 5h, i). G-ratios from cuprizone-treated *miR-145*^*+/+*^ corpus callosum were 0.77 ± 0.003 and 0.79 ± 0.005 at 12 weeks cuprizone and 5 weeks recovery, respectively, suggesting that the small increase in myelinated axon number observed between these two time points in *miR-145*^*+/+*^ animals may be due to a minor amount of remyelination.

Upon qRT-PCR assessment of miR-145-5p expression in chronically demyelinated *miR-145*^*+/+*^ animals, we found that miR-145-5p expression was upregulated relative to age-matched controls following 12 weeks cuprizone exposure, and this differential upregulation is maintained even after 5 weeks recovery (Fig. 5j). This is in contrast with acutely demyelinated mice and in line with our findings in human MS chronic lesions, suggesting that increased miR-145-5p expression is a pathological feature of chronic but not acute demyelination.

**Fig. 6:**
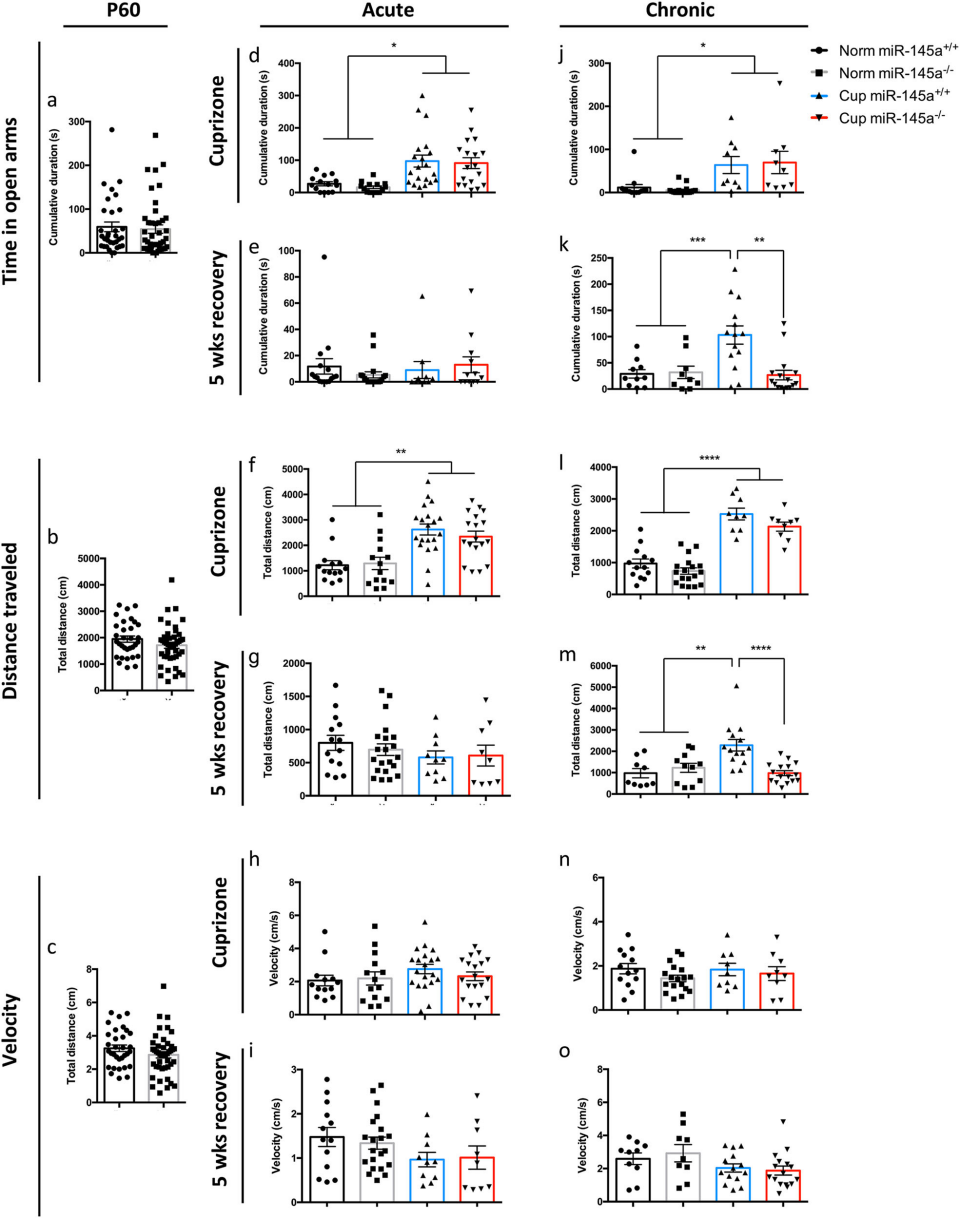
Loss of miR-145 restores normal anxiogenic response after recovery from chronic cuprizone exposure. Elevated plus maze (EPM) was used to assess anxiety behaviours in *miR-145*^*+/+*^ and *miR-145*^*–/–*^ animals on normal (Norm) or cuprizone (Cup) diet at P60 (**a**–**c**), with acute cuprizone exposure (**d**–**i**) and with chronic cuprizone exposure (**j**–**o**). **a**, **d**, **e**, **j**, **k** Time spent in open arms of the EPM. **b**, **f**, **g**, **l**, **m** Distance travelled throughout the trial in the EPM. **c**, **h**, **i**, **n**, **o** Average velocity throughout the trial in the EPM. *N* = 9–44, *p < 0.05, **p < 0.01, ***p < 0.001, ***p < 0.0001, one-way ANOVA with Tukey’s *post hoc*. All data represent mean ± SEM. Legend applies to all panels.

**Fig. 7:**
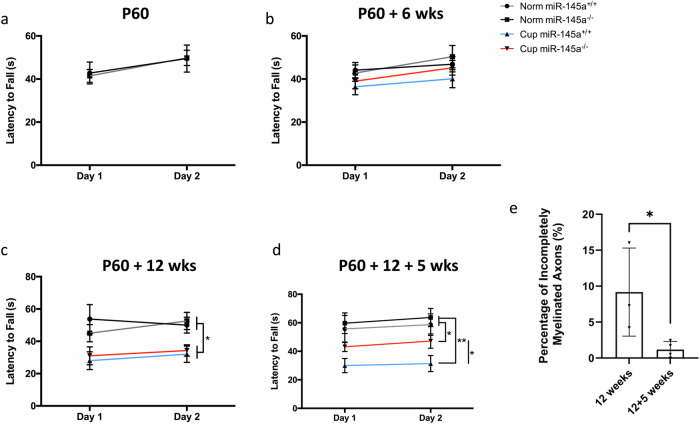
Motor coordination deficit is partially restored with miR-145 loss following recovery from chronic cuprizone exposure. Rotarod testing was used to assess motor coordination in *miR-145*^*+/+*^ and *miR-145*^*–/–*^ animals on normal (Norm) or cuprizone (Cup) diet at P60 (**a**), P60 + 6 weeks (wks) (**b**) normal or cuprizone diet, P60 + 12 wks (**c**) normal or cuprizone diet, and P60 + 12 + 5 wks (**d**) normal diet or 12 wks cuprizone diet followed by 5 wks normal diet. Latency to fall was measured for 4 trials on 2 consecutive days. *N* = 6–13, *p < 0.05, **p < 0.01, one-way ANOVA with Tukey’s *post hoc*. **e** Quantification of percentage of incompletely myelinated axons in *miR-145*^*–/–*^ animals on cuprizone diet for 12 weeks, or cuprizone diet for 12 wks followed by 5 wks normal diet. *N* = 3, *p < 0.05, Student’s *t* test. Values represent mean ± SEM.

To determine whether the substantive remyelination observed in *miR-145*^*−/−*^ animals following chronic cuprizone exposure was sufficient to result in functional recovery, we conducted behavioural assessments in both normal and demyelinated *miR-145*^*+/+*^ and *miR-145*^*−/−*^ animals. The neurological consequences of cuprizone-mediated demyelination are measurable as altered anxiogenic behaviours and motor coordination deficits. To test these in our acute and chronic cuprizone models, we utilized the elevated plus maze (EPM) and rotarod to measure changes in anxiety and motor coordination, respectively. Anxiogenic responses are altered in cuprizone-treated animals, usually resulting in reduced preference to remain in dark, enclosed spaces and increased desire to explore when exposed to unfamiliar environments. No inherent differences in anxiety between *miR-145*^*+/+*^ and *miR-145*^*–/–*^ animals were observed at the start of cuprizone treatment at P60, or in age-matched healthy controls during the time courses of acute or chronic cuprizone treatment (Fig. 6a–o). After 6 weeks cuprizone exposure, reduced anxiogenic responses were detectable in both *miR-145*^*+/+*^ and *miR-145*^*−/−*^ animals to similar degrees, as both spent increased time in the open arms of the EPM (Fig. 6d). Further, cuprizone-treated animals spent more time exploring the maze, as evidenced by greater distance travelled (but no change in velocity) during the timed 10-min trial when compared to age-matched controls (Fig. 6f, h). These alterations in acutely treated cuprizone animals were returned to normal after 5 weeks recovery in both *miR-145*^*+/+*^ and *miR-145*^*–/–*^ animals, consistent with the remyelination observed in the corpus callosum (Fig. 6e, g, i). Increased time in the EPM open arms and greater distance travelled were maintained in both *miR-145*^*+/+*^ and *miR-145*^*−/−*^ animals after chronic cuprizone exposure (Fig. 6j, l). This suggests that in *miR-145*^*−/−*^ animals, the extent of remyelination observed above was not sufficient to negate the neurological deficits incurred at this stage with respect to anxiety. However, after 5 weeks recovery from chronic treatment, *miR-145*^*–/–*^ animals reverted to healthy control anxiogenic behaviours, both for time spent in the EPM open arms and distance travelled, while *miR-145*^*+/+*^ animals retained their acquired abnormal anxiogenic responses (Fig. 6k, m, o). With respect to motor coordination, animals were assessed by measuring time taken to fall from a rotating rod programmed to increase rotations per minute (rpm) at a steady state over time. Again, no inherent differences were observed in healthy *miR-145*^*+/+*^ and *miR-145*^*–/–*^ animals at any time point assessed (Fig. 7a–d). Further, no motor deficits were detectable using this measurement even after 6 weeks cuprizone exposure (Fig. 7b); thus, acutely-treated animals were not assessed at recovery using this measure. After 12 weeks cuprizone treatment, motor coordination deficits were detectable and both *miR-145*^*+/+*^ and *miR-145*^*−/−*^ mice exhibited similar reduced latency to fall (Fig. 7c); this again suggests that the remyelination observed in *miR-145*^*−/−*^ animals at this time point was insufficient to correct motor coordination deficits caused by chronic cuprizone exposure. After 5 weeks recovery, however, *miR-145*^*–/–*^ animals did exhibit partial recovery of motor coordination with latency to fall significantly increased over *miR-145*^*+/+*^ cuprizone animals (Fig. 7d). To address why *miR-145*^*–/–*^ mice treated with cuprizone for 12 weeks did not demonstrate recovery in the behavioural tests (Figs. 6, 7c), despite the increased remyelination observed (Fig. 5b, d), we assessed the quality of the myelin and compared it to that in *miR-145*^*–/–*^ mice treated with cuprizone for 12 weeks with 5 weeks recovery. We noted that there was a significantly higher percentage of incompletely myelinated axons in the 12 weeks cuprizone treated cohort compared to the 12 weeks cuprizone with 5 weeks recovery cohort (9.17% vs. 1.18%; *p* < 0.05; Fig. 7e; qualitatively observed in Fig. 5b, c). This may partly explain the difference in functional recovery between the two cohorts.

### Overexpression of miR-145-5p leads to severe OL differentiation deficits and OL death

**Fig. 8:**
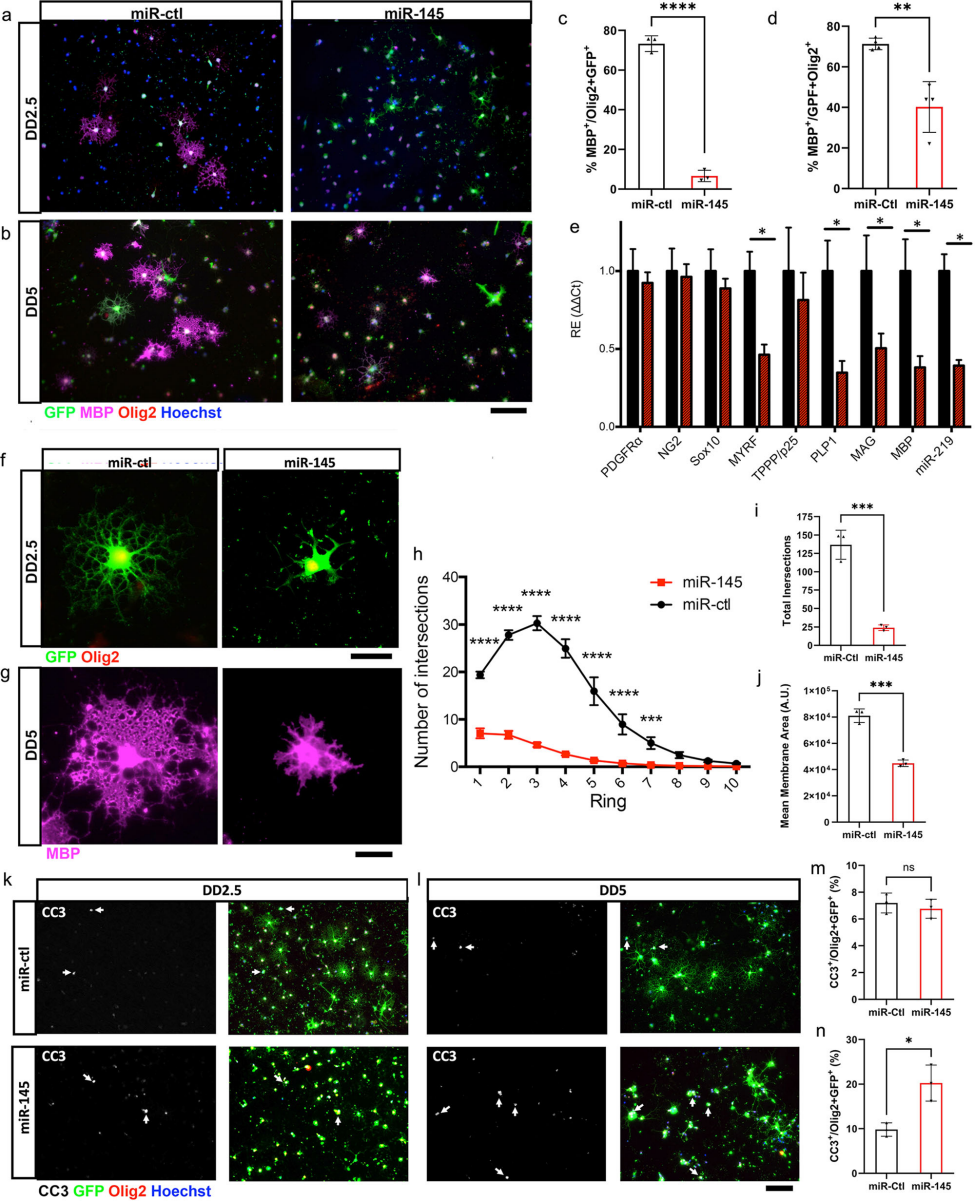
Overexpression of miR-145 limits myelin protein expression and severely stunts morphological differentiation of OLs. Fluorescent micrographs of lentivirus transduced OLs on differentiation day 2.5 (DD2.5; **a**) and differentiation day 5 (DD5; **b**). Left panels—miR-ctl; right panels—miR-145. GFP (green) indicates transduced cells; additional staining for myelin basic protein (MBP; magenta), Olig2 (red), counterstained with Hoechst (blue). Scale bar = 100 µm. Quantification of % MBP^+^/GFP^+^ and Olig2^+^ cells at DD2.5 (**c**) and DD5 (**d**). *N* = 4; **p < 0.01, ****p < 0.0001, Student’s *t* test. **e** Gene expression of major markers of OL differentiation at DD2.5 by qRT-PCR. *N* = 4, *p < 0.05, Student’s *t* test adjusted for multiple propagations. Analysed by ΔΔCt (**f**, **g**). Confocal micrographs of single OLs on DD2.5 (**f**) showing GFP expression and stained for Olig2, and DD5 (**g**) stained for MBP. Left panels—miR-ctl; right panels—miR-145. Scale bars = 50 µm. **h** Sholl analysis of miR-ctl and miR-145 DD2.5 OLs. *N* = 4; ***p < 0.001, ****p < 0.0001, two-way ANOVA with Sidak *post hoc* test. **i** Quantification of total branch intersections from Sholl analysis of miR-ctl and miR-145 DD2.5 OLs. *N* = 4; ***p < 0.001, Student’s *t* test. **j** Quantification of total membrane area of miR-ctl and miR-145 DD2.5 OLs. *N* = 4; ***p < 0.001, Student’s *t* test. Fluorescence micrographs of DD2.5 (**k**) and DD5 (**l**) OLs. Left panels—cleaved-caspase 3 (CC3; grey); right panels—merge with GFP and Olig2, counterstained with Hoechst. Scale bar = 100 µm. White arrows indicate CC3^+^/GFP^+^/Olig2^+^ cells. Quantification of % CC3^+^/GFP^+^ and Olig2^+^ OLs on DD2^.^5 (**m**) and DD5 (**n**). *N* = 4; ns not significant, *p < 0.05 Student^’^s *t* test. All values represent mean ± SEM.

Given our previous work showing that miR-145-5p expression in rat OPCs downregulated OL differentiation^21^, we reasoned that the pathological upregulation of miR-145-5p observed in the chronic demyelination experiment might inhibit OL maturation. To test this, we employed lentiviral transduction in primary rat OLs to overexpress miR-145 (Supplementary Fig. 5a) and assessed both molecular and morphological differentiation of OLs at intermediate (DD2.5) and late differentiation (DD5) time points. Indeed, both aspects of OL differentiation suffered deficits with miR-145 overexpression. The proportion of MBP^+^ OLs was significantly decreased on DD2.5 from 73.3% in scrambled control OLs (miR-ctl) to 6.6% in miR-145 overexpressing OLs (miR-145), and on DD5 from 77.3% in miR-ctl to 40.2% in miR-145 OLs (Fig. 8a–d). Investigation of major markers of OL differentiation revealed strong loss of expression of myelin genes *MYRF, PLP1, MAG*, and *MBP* as well as pro-differentiation miRNA miR-219 at DD2.5 (Fig. 8e). Morphologically, miR-145 OLs were severely stunted. Branching quantification by Sholl analysis of DD2.5 OLs showed few branches with very limited complexity in miR-145 cells when compared to miR-ctl (Fig. 8f, h, i). By DD5, sufficient OLs reached the milestone of MBP expression to assess membrane area, but of those miR-145 OLs able to produce MBP their membrane area was reduced by half that of miR-ctl Ols (Fig. 8g, j). Finally, the generally unhealthy appearance of miR-145 OLs prompted us to conduct an evaluation of cell death. Based on cleaved-caspase 3 (CC3) staining, caspase-mediated apoptosis occurred at a similar rate in miR-ctl and miR-145 OLs at DD2.5 (Fig. 8k, m); however, by DD5, the proportion of CC3^+^ OLs was more than double in miR-145 cells when compared to miR-ctl cells (Fig. 8l, n). Thus, overabundance of miR-145-5p not only rigorously limits OL differentiation ability, but prolonged exposure also promotes OL death.

**Fig. 9:**
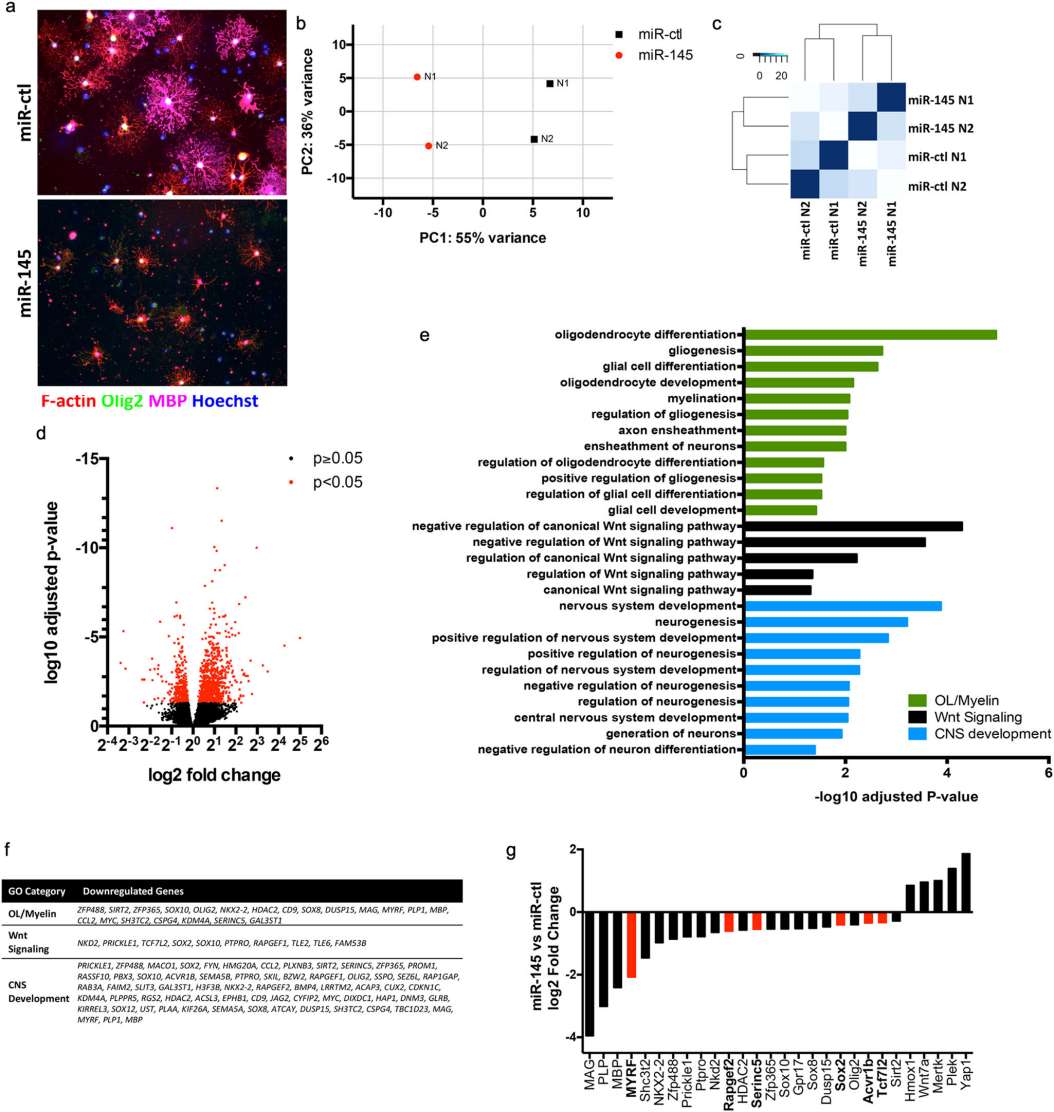
RNAseq reveals downregulation of multiple factors in critical OL differentiation and myelination pathways with overexpression of miR-145. **a** Fluorescence micrographs of primary differentiating OLs transfected with scrambled control (miR-ctl; left panel) or miR-145 mimic (miR-145; right panel) on differentiation day 5. Cells are stained for F-actin (red), Olig2 (green) and MBP (magenta) and counterstained with Hoechst. **b** Principal component analysis (PCA) of all mapped transcripts for all samples. Plot shows two-dimensional comparison of *N* = 2 for miR-ctl and miR-145 calculated using the DESeq2 plotPCA function and rlog-transformed count data. **c** Heat map displaying clustering analysis for *N* = 2 for miR-ctl and miR-145. **d** Volcano plot of log2 fold change versus log10 adjusted *p* value for all mapped transcripts after filtering for miR-145 relative to miR-ctl. Fold change was calculated using DESeq2, *p* value was adjusted using the Benjamini-Hochberg method. **e** Significantly enriched gene ontology (GO) terms from genes differentially downregulated in miR-145 OLs relative to miR-ctl, sorted by overarching category. **f** All differentially downregulated genes identified by GO term analysis overarching categories. **g** Validation of selected significantly differentially expressed genes by qRT-PCR. *N* = 3, *p < 0.05, **p < 0.01, ***p < 0.001, ****p < 0.0001, Student’s *t* test adjusted for multiple propagations. Analysed by ΔΔCt, expressed as log2 fold change relative to miR-ctl. Genes in bold/red bars indicate confirmed/putative targets of miR-145-5p.

In addition to our analysis of myelin gene expression (Fig. 8e), to determine how miR-145-5p stunts OL differentiation at the molecular level, we performed RNAseq to assess the transcriptomic response to overexpression of miR-145. Sequencing was performed in two replicates for each of miR-145 and miR-ctl on DD5. For this experiment, we treated cells with miR-145-5p mimics as an alternative approach to lentiviral transduction, which similarly resulted in significant miR-145-5p upregulation and the same differentiation deficits as described above (Fig. 9a, Supplementary Fig. 5b). Reads processed by RNAseq exceeded 40 million in all samples, with ~80% mapping coverage for each. Raw data variance was assessed by principal component analysis (PCA) and hierarchical clustering; differences between miR-ctl and miR-145 samples explained 56% of the total variance, while intra-sample variance explained 36% of the total variance (Fig. 9b). Despite the intra-sample variance, replicates clearly clustered together based on condition (Fig. 9c). Of 16,129 genes identified by mapping, 1086 showed significant differential expression in miR-145 OLs relative to miR-ctl, with 330 downregulated genes and 756 upregulated genes (Fig. 9d). Significantly altered genes were divided into two sets based on upregulation and downregulation, and these two data sets were each subjected to gene ontology enrichment analysis. The top three significantly enriched gene ontology categories arising from downregulated factors were oligodendrocyte differentiation, negative regulation of canonical Wnt signalling, and nervous system development – all of which promote OL differentiation. In fact, multiple significantly enriched additional categories kept with these themes, resulting in 12 categories generally representing OLs/myelin formation, 5 representing regulation of Wnt signalling, and 10 representing CNS development (Fig. 9e, Supplementary Data 2). Specific genes from these enriched pathways included some of those previously found to be upregulated with miR-145-5p knockdown (such as *MYRF*, *MAG*, *MBP*, and *PLP*), and expanded upon those to include others known to be critical to OL differentiation such as *ZFP488*, *ZFP365, SOX10, DUSP15, Tcf7l2*, and *Prickle1*, amongst others (Fig. 9f). Of the genes identified by gene ontology enrichment, six were confirmed or putative targets of miR-145-5p, suggesting initiating points for the downstream effects observed with miR-145-5p overexpression (Fig. 9g, bold genes/red bars). Upregulated genes were broadly categorized as being involved with damage response, extracellular matrix organization and ion homoeostasis, likely to be compensatory due to the massive dysregulation of differentiation genes (Supplementary Fig. 6, Supplementary Data 3). Selected genes identified by gene ontology enrichment, including the miR-145-5p targets, were validated by qRT-PCR, confirming downregulation of a host of critical differentiation factors as well as upregulation of several genes with known roles in differentiation in OLs overexpressing miR-145 (Fig. 9g).

### Loss of miR-145 alters glial cell numbers within the corpus callosum following chronic toxic demyelination

**Fig. 10:**
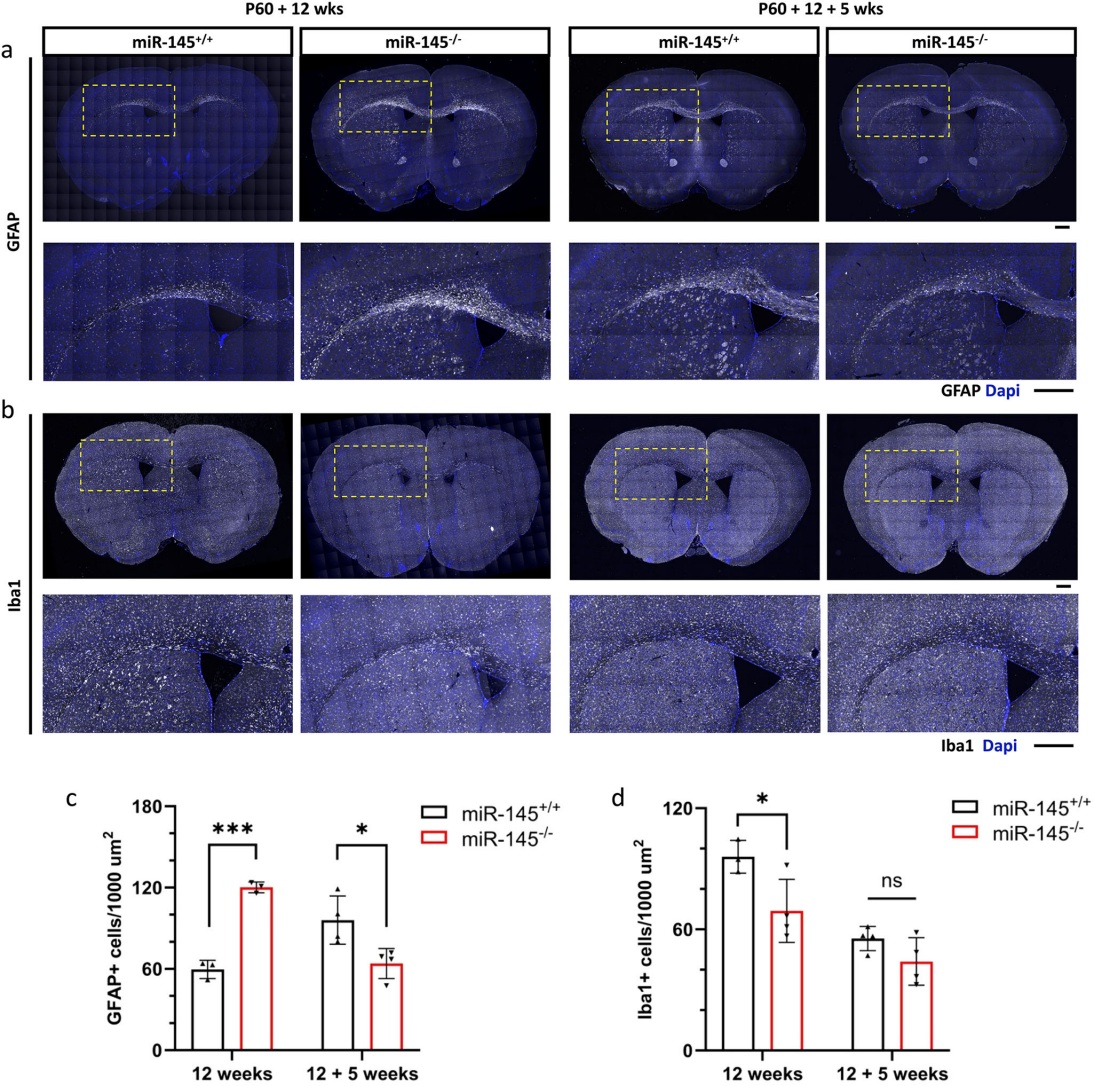
Loss of miR-145 differentially affects astrocyte and microglia recruitment to corpus callosum following chronic cuprizone exposure. **a**, **b** Fluorescence micrographs of coronal sections of corpus callosum in *miR-145*^*+/+*^ and *miR-145*^*–/–*^ animals after 12 weeks of cuprizone exposure (left panels) or 12 weeks cuprizone exposure followed by 5 weeks recovery on normal diet (right panels). Tissues were stained with GFAP (grey) (panels in **a**) or Iba1 (grey) (panels in **b**), and counterstained with Dapi (blue). Yellow boxes in each upper panel of (**a**) and (**b**) show zoomed area in corresponding lower panel. Scale bars = 500 µm. Quantifications of the number of GFAP+ cells per 1000 µm^2^ (**c**) and number of Iba1+ cells per 1000 µm^2^ (**d**) in the corpus callosum of *miR-145*^*+/+*^ and *miR-145*^*–/–*^ animals after 12 weeks of cuprizone exposure or 12 weeks cuprizone exposure followed by 5 weeks recovery on normal diet. *N* = 3–4, ns not significant, *p < 0.05, ***p < 0.001, Student’s *t* test. All values represent mean ± SEM.

In the CNS, myelination is largely regulated by oligodendrocytes. However, other glial cells, including astrocytes and microglia, can also contribute to this process. We performed immunohistochemical studies on sections of corpus callosum from wild-type and *miR-145*^*–/–*^ animals following 12 weeks cuprizone exposure or 12 weeks cuprizone exposure followed by 5 weeks of recovery (Fig. 10). The total number of astrocytes (GFAP^+^ cells) was nearly doubled in the corpus callosum of *miR-145*^*–/–*^ animals after 12 weeks on cuprizone compared to wild-type animals (Fig. 10a, c). However, after an additional 5 weeks of recovery, *miR-145*^*–/–*^ animals displayed a decrease in these cells relative to wild-type animals (Fig. 10a, c). In comparison, the presence of microglia in *miR-145*^*–/–*^ animals was significantly lower than in wild-type animals after 12 weeks of cuprizone treatment as determined by quantification of Iba1^+^ cells (Fig. 10b, d). After an additional 5 weeks of recovery, the microglia number in the *miR-145*^*–/–*^ animals were comparable to those in wild-type animals (Fig. 10b, d). Collectively, these data suggest that miR-145 loss impacts glial cell response in chronic cuprizone-mediated demyelination, and consequently could also impact remyelination and recovery in this model.

## Discussion

A host of dysregulated miRNAs have been identified in lesion tissue taken from both RRMS and progressive MS brains, but the potential roles of these miRNAs in disease pathogenesis remain poorly characterized. MiR-145-5p was previously identified by microarray as overabundant in chronic MS lesion tissue by Junker et al.^22^, and we recently characterized miR-145-5p as an anti-differentiation/pro-proliferation gatekeeper specifically in oligodendrocytes^21^. Here, we validated that miR-145-5p level is elevated in chronic inactive MS lesion tissue and further employed a miR-145 knockout model to characterize the effects of miR-145 loss in developmental myelination and in remyelination using both the acute and chronic cuprizone demyelination paradigms.

When primary OPCs were isolated from wild-type and miR-145 deficient animals and differentiated into OLs in vitro, we demonstrated a subtle acceleration of the appearance of myelin markers MAG and MBP, as well as of the formation of compact myelin membrane sheets. Despite this, in miR-145 knockout mice we were unable to detect evidence of accelerated myelination during development in vivo. Other factors may be at play which prevent the untimely differentiation of OPCs in vivo, since spontaneous differentiation is not necessarily beneficial to the developing CNS. OPCs are required to migrate extensively and often proliferate in situ to meet the myelination demands of specific compartments and under specific circumstances, such as in the event of demyelination – premature differentiation would prevent fulfilment of these functions. Additionally, OL differentiation and myelination are achieved in a drastically shorter timeframe in vivo than in vitro, the former likely being achieved in a matter of hours while the latter requires days^25^. This raises the possibility that OL differentiation is indeed accelerated with loss of miR-145 in vivo, but that the window of difference is miniscule and may not be biologically relevant. Further, any difference is clearly transient, since in corpus callosum from mature adults from P60 and several time points up to and including P179 (normal animals at P60 + 12 + 5 weeks) showed similar numbers of myelinated axons at every assessed stage. Regulation of myelin thickness is also normal with loss of miR-145, as g-ratios were similar at those same assessed time points. Upon induction of demyelination following an acute course of 0.2% cuprizone administration, the extent of remyelination appeared similarly unaffected as after 5 weeks of recovery on normal diet, numbers of myelinated axons in the corpus callosum were not different between *miR-145*^*+/+*^ and *miR-145*^*−/−*^ animals and functional recovery was equally efficient in both. Again, this does not preclude the presence of differences that may have occurred at an earlier time point, but by the same token any changes to the course of remyelination were transient.

In contrast with the acute demyelination paradigm, chronic demyelination revealed differences between wild-type and miR-145 deficient animals. Remyelinated axons were apparent in miR-145 deficient animals following 5 weeks of recovery after 12 weeks of cuprizone exposure, as well as at the end of the cuprizone course prior to their return to normal diet, suggesting that remyelination occurred even as the animals were maintained on the cuprizone diet. While this was initially perplexing in comparison with the acute treatment which showed no impact on remyelination, we further discovered in wild-type animals that while miR-145-5p expression is unchanged with acute cuprizone administration, it is significantly upregulated both at 12 weeks cuprizone and after recovery relative to untreated animals. These findings suggest that loss of normal physiological levels of miR-145-5p in vivo does not seem to enhance remyelination under circumstances when remyelination already proceeds normally. However, prevention of miR-145-5p pathological upregulation, achieved here via its loss in the knockout mouse, can allow remyelination to proceed when it otherwise cannot. This in turn implies that upregulation of miR-145-5p may in itself be an inhibitor to remyelination, supported by our findings in primary OLs where overexpression of miR-145-5p severely stunts differentiation and even leads to OL death, as well as by the fact that during the normal course of OL differentiation, miR-145-5p is strongly downregulated as OPCs transition to OLs^21,26^. These effects on OL differentiation and survival when miR-145-5p is overexpressed are driven by reduced downstream expression of multiple known OL differentiation factors such as *MYRF* and its downstream targets *MAG, PLP, MBP* and *Dusp15*, other pro-differentiation factors *Zfp488, Zfp365, Gpr17, Tcf7l2, Sox2* and negative regulators of Wnt signalling *Prickle1* and *Nkd2*, amongst others. Several of these genes are targets of miR-145-5p, including Sox2, Tcf7l2 and MYRF^21,27–29^. Intriguingly, Tcf7l2 is upstream of MYRF, and Sox2 is in turn upstream of Tcf7l2, suggesting that miR-145-5p may exert multi-level control over critical regulatory networks involved in OL differentiation^28,29^. When considering genes upregulated in response to miR-145-5p overexpression, most are categorized as a response to damage or wounding and organization of the extracellular matrix – all consistent with cells undergoing stress, as OLs induced to differentiate whilst overexpressing miR-145 clearly are. Some interesting factors which may be upregulated to compensate for stalled differentiation pathways include validated genes *Mertk, Hmox1*, *Yap1* and *Plek*. Mertk and Hmox1 are typically upregulated in OLs as a protective response to inflammatory stress, while Yap1 is involved in promoting branch extension in the face of mechanical stress^30–32^. In addition, mutations in *Mertk* and *Plek* are associated with increased MS risk and may thus represent a reduced ability of OLs to respond to stress in that context, making them more vulnerable to inflammatory attack^33,34^.

The upregulation of miR-145-5p is an important distinction between the acute and chronic demyelination paradigms. The chronic cuprizone model of demyelination also results in the build-up of CSPGs and FGF2, both of which are inhibitory to remyelination via prevention of OL differentiation^20,23^. Upregulation of these factors along with miR-145-5p is mirrored in progressive MS lesions, also reticent to remyelination^22^. Models such as this, that recapitulate accumulation of inhibitory factors found in progressive lesions, are critical to the study of remyelination therapies in ways we are only coming to appreciate now – not only in the study of the effects of these factors on remyelination, but also in the upstream signalling pathways that lead to their overabundance in that context. Several pro-myelinating factors have been identified in recent years such as clemastine, benztropine, quetiapine, miconazole and clobetasol, all of which promote OPC differentiation in vitro and promote myelination in vivo under healthy conditions^35–37^. While these initially generated considerable excitement as potential therapeutics for progressive MS, they have thus far failed to promote OL differentiation or myelin production when faced with even a single inhibitory challenge from amongst the plethora of those found in a progressive MS lesion. This was thoroughly illustrated in a study by Keough et al., in which these small molecules could not push OLs to overcome differentiation deficits to any degree when cultured with CSPGs in vitro; the only path to success was to enzymatically remove CSPGs^38^. This highlights the need not only to further our understanding of the mechanisms behind how these factors inhibit OL differentiation, but also how to promote OL differentiation specifically in the face of these inhibitors. Only then can truly relevant therapeutic targets for progressive MS be identified, since the promotion of positive cues without a concurrent focus on the removal of inhibitory cues is obviously not sufficient to promote OL differentiation and remyelination in this context.

When considering the progressive MS lesion, the hallmark that sets it apart from a RRMS lesion is its inability to regenerate lost myelin. Remyelination requires recruitment of OPCs to the lesion site, and their subsequent differentiation and remyelination of denuded axons in a manner that largely recapitulates the process of developmental myelination. The population of OPCs and OLs particularly at chronic lesions exhibits some heterogeneity and includes both OPCs and early differentiating OLs^15,16,39^. Importantly the latter are largely characterized as OLs that have entered the differentiation program but are arrested prior to achieving MYRF positivity^16^. An overexpression of miR-145 may have two possible roles in this case – first, our previous work showing the ability of miR-145 to potentiate proliferation suggests that it may play a role in OPC proliferation at the chronic lesion site^21^. Secondly, based on our findings here, an overabundance at the lesion site may contribute to the arrest of early OLs during the differentiation program by blocking the expression of MYRF and its upstream mediators Sox2 and Tcf7l2 as well as dysregulating Wnt signalling such that they cannot mature to completion. In this sense, miR-145-5p may contribute to remyelination failure in two ways – by preventing both the transition of OPCs to OLs, as well as the transition from differentiating OL to myelinating OL. Future studies on profiling in chronic lesions from MS patients such as the expression of the upstream mediators Sox2 and Tcf7l2, as well as some of the other factors identified in our cell culture RNA-seq experiment, could be pursued.

While the source of miR-145-5p at the site of chronic demyelination remains unclear, it may be OPC-intrinsic, -extrinsic, or both. Additional work is needed to understand the contributing sources of miR-145 dysregulation within the lesion environment. Abnormal intrinsic expression may be driven by p53, which is upregulated in OPCs and OLs in active MS lesions and drives miR-145 expression via direct interaction with the miR-145 promoter^40,41^. Uptake of exogenous miR-145 may also occur, as its expression is common to other cell types present in the lesion, which may secrete miRNA-laden exosomes subsequently taken up by OPCs incoming to the demyelinated area^42,43^. In this sense, though OLs are strongly implicated in the effects of overexpression of miR-145-5p at the lesion site, other cell types may also be at play. Both reduced inflammation and neuroprotection are possible outcomes with the loss of miR-145; though miR-145 knockout did not attenuate demyelination, as both wild-type and miR-145 deficient animals were equally demyelinated by six weeks exposure to cuprizone, altered responses to chronic demyelination may be present in astrocytes, neurons and/or microglia due to miR-145 loss. Indeed, we have observed altered glial cell response in the chronic cuprizone mediated demyelination in *miR-145*^*−/−*^ mice. Not unlike in OLs, miR-145 overexpression has been shown to stunt branching in astrocytes and neurite extension in neurons, and also promotes pro-inflammatory functions in microglia^44–46^; additional work must therefore be undertaken to understand other cell-type specific implications of the pathological upregulation of miR-145-5p with chronic demyelination and how negating this upregulation aids in overcoming remyelination failure.

In conclusion, when taken collectively, our work has demonstrated that miR-145 loss is perhaps not relevant as a strategy to enhance myelination or remyelination under circumstances where its expression remains at normal levels, but is sufficient to promote remyelination and functional neurological recovery following chronic demyelination where we report that miR-145-5p expression is pathologically upregulated. Like the overabundance of CSPGs and FGF2 with chronic demyelination, the dysregulation of miR-145-5p is established as an important factor that distinguishes remyelination-reticent lesions from those that spontaneously remyelinate. From an OL perspective, we show that excessive miR-145-5p severely limits OL differentiation and contributes to caspase-mediated apoptosis of OLs, underpinned by reduced expression of critical differentiation and myelination pathways and despite the upregulation of multiple protective genes. These are important findings as miR-145-5p is also upregulated in chronic lesions in progressive MS. This work begins to unravel not only how miR-145-5p may contribute to remyelination failure in human disease, but also highlights it as a potential therapeutic target to alleviate the OL differentiation block observed in the progressive MS lesion microenvironment.

## Methods

### Human tissues

Human brain tissue was obtained from patients diagnosed with clinical and neuropathological MS according to the revised 2010 McDonald’s criteria^47^. Tissue samples were collected from healthy donors and MS patients with full ethical approval (BH07.001) and informed consent from the Centre de recherche du Centre hospitalier de l’Université de Montréal ethics committee. All ethical regulations relevant to human research participants were followed. Autopsy samples were flash-frozen, and lesions classified using Luxol Fast Blue/Hematoxylin & Eosin staining and Oil Red O staining as previously published^48,49^.

Patient information is summarized in Table 1. Tissues representing active lesions, chronic inactive lesions and normal appearing white matter (NAWM) were collected from four SPMS patients and one RRMS patient, along with white matter (WM) from four healthy controls (HCs).

### Animals

*MiR-145*^*−/−*^ mice generated on the C57BL/6 background were generously provided by Dr. Eric Olson and are described in Xin et al.^50^. Animals were sacrificed at P0-P2 for primary cell culture, at P0, P9, P15, P30 and P60 for developmental in vivo analyses, and at various time points following treatment with cuprizone, as indicated. All animals were provided water and chow *ad libitum* and maintained on a 12:12 h light cycle.

The University of Ottawa Animal Care Committee approved all experimental protocols involving animals. The protocols conformed to or exceeded those defined in the Canadian Council on Animal Care’s Guide to the Care and Use of Experimental Animals, and the Animals for Research Act. We have complied with all relevant ethical regulations for animal use.

### Cell culture

Primary mouse OPCs were isolated and cultured as per O’Meara et al^51^. First, mixed glial cultures (MGCs) were generated from P0-P2 *miR-145*^*+/+*^ and *miR-145*^*−/−*^ mouse pups. Cortices were dissociated with papain, and the resulting suspension was plated in Dulbecco’s modified Eagle medium (DMEM; Wisent) containing 10% foetal bovine serum, 1% Glutamax (Gibco), 5 μg/mL insulin (Sigma) and 0.33% penicillin/streptomycin (Gibco). These cells were plated in poly-L-lysine-coated filter cap flasks for 6 days and maintained at 37 °C and 8.5% CO_2_. The medium was then supplemented with 5 μg/mL insulin (Sigma), and cultures were moved to 5% CO_2_ for an additional 3–4 days. Cultures were then shaken overnight at 220 rpm, and medium containing suspended OPCs was removed and further enriched by separating out contaminating glial cells by differential adhesion as above. The OPCs were then plated in SATO medium with 0.5% foetal bovine serum, 1% Glutamax, 5 μg/mL insulin, 50 μg/mL holo-transferrin (Sigma), 50 ng/mL ciliary neurotrophic factor (CNTF; Serotec) and 0.33% penicillin/streptomycin on coverslips dual-coated with poly-L-lysine and kept at 37 °C and 5% CO_2_ until collection.

Immortalized oligodendrocyte progenitor *Oli-neu* cells were incubated in SATO medium with 1% horse serum in DMEM (Wisent) at 37.5 °C and 10% CO_2_ as previously described^52^. Medium was replaced every 2–3 days. Cells were plated on poly-L-lysine coated plates (poly-L-lysine; Sigma). To differentiate cells, 1 mM dbcAMP (Sigma) was added to the medium. To passage cells, plates were incubated for 1 min with 0.05% trypsin (Invitrogen) at 37.5 °C, followed by trypsin deactivation with 10% horse serum in DMEM. Detached cells were spun at 1200 rpm for 3 min and then replated in SATO medium as above.

Primary rat OPCs were isolated and cultured as per Chen et al.^53^. First, MGCs were generated by dissecting cerebral cortices from P1-P3 Sprague-Dawley rat pups. Cortices were digested for 15 min at 37 °C in 1% trypsin (Sigma-Aldrich) with 10.7 µ/mL DNase I (Sigma-Aldrich) in Hank’s Balanced Salt Solution (HBSS). Digested tissue was triturated in MGC medium made up of Dulbecco’s Modified Eagle Medium (DMEM; Wisent) with 20% FBS, 2 mM L-glutamine (Invitrogen), 50 units/mL penicillin and 50 µg/mL streptomycin (Thermo-Fisher). Triturated tissue suspensions were allowed to settle on ice for 10 min followed by filtration through a 70 µm strainer and were then incubated in MGC medium on poly-L-lysine (PLL)-coated plates at 37 °C and 5% CO_2_ for 7-10 days with 75% MGC medium changes every 2-3 days. To obtain enriched OPC cultures, MGCs were shaken for 18–20 h at 37 °C and 5% CO_2_. Medium containing suspended OPCs was allowed to settle on coated culture plates (Corning) for 30 min at 37 °C and 5% CO_2_ to remove contaminating cells by differential adhesion. OPCs were pelleted from suspension by centrifugation at 100 × *g* for 10 min and were then resuspended in appropriately supplemented SATO medium. SATO medium comprised 5 ug/mL insulin, 1 µg/mL bovine serum albumin (BSA), 0.05 µg/mL apo-transferrin, 0.03 µg/mL sodium selenite, 0.25 µg/mL D-biotin and 0.01 µg/mL hydrocortisone (all reagents from Sigma-Aldrich) in DMEM (Wisent) with 2 mM L-glutamine (Invitrogen) and 50 units/mL penicillin and 50 µg/mL streptomycin (Thermo-Fisher). OPCs were plated in SATO medium supplemented with 0.01 µg/mL basic fibroblast growth factor (FGF2; Millipore) and 0.01 µg/mL platelet-derived growth factor AA (PDGFAA; Millipore), and 50% media changes were done every 2 days. OPCs being differentiated into Ols were plated in SATO medium supplemented with 15 nM triiodothyronine (T3; Sigma-Aldrich), 0.01 µg/mL ciliary neurotrophic factor (CNTF; Peprotech) and 5 ng/mL N-acetyl L-cysteine (Sigma-Aldrich). To transition OPCs to Ols after plating, a 50% media change was done using 2X differentiation medium.

### Immunofluorescence

Cells were fixed in 3% paraformaldehyde (PFA) for 15 min, washed 2 x with PBS, followed by permeabilization in 0.1% Triton X-100 in PBS for 5 min. After washing again 3 x with PBS, cells were incubated in blocking medium (10% goat serum in PBS) for ~1 h at 4 °C. Incubation with primary antibody was done in 10% goat serum overnight at 4 °C, followed by 3 x washes with 1x PBS and 2-3 h incubation with secondary antibody at 4 °C. F-actin was visualized using rhodamine-conjugated phalloidin (Invitrogen) 1:50 in blocking serum for ~2 h at 4 °C. Nuclear staining was achieved using Hoechst 1:1000 in PBS for ~5 min at room temperature. Coverslips were mounted in Dako Fluorescence Mounting Medium. Primary antibodies were diluted in blocking serum at the following concentrations: rabbit anti-Olig2 1:500 (EMD Millipore); rat anti-MBP 1:100 (AbD Serotec), mouse anti-MAG 1:50 (EMD Millipore), rabbit anti-cleaved caspase 3 1:200 (Asp175; Cell Signalling Technology). Appropriate AlexaFluor secondary antibodies (Invitrogen) were diluted 1:200 in blocking serum.

### RNA isolation and qRT-PCR

For all samples, total RNA was extracted using the RNeasy Mini Kit (Qiagen), according to the manufacturer’s protocol. Frozen human tissue samples, fresh mouse forebrain/midbrain tissue, as well as *Oli-neu* or primary OPCs/OLs grown in a 6-well plate were lysed using 350 µl RLT lysis buffer per sample. Lysis buffer was added directly to frozen tissue or washed cells, and lysate was then placed in a Qiashredder column (Qiagen) and spun at maximal speed for 2 min. Lysate was then mixed 1:1 with 70% ethanol in RNase-free H_2_O, transferred to the RNeasy spin column, and centrifuged at maximal speed for 25 s. Eluate was discarded, and the column was washed with 700 µl RW1 buffer by spinning at maximal speed for 25 s. Eluate was again discarded, and the column was washed with 500 µl RPE buffer at maximal speed for 25 s. The columns transferred to a clean collection tube and washed a second time with 500 µl RPE for 2 min. Columns were then placed in RNase-free microfuge tubes, and RNA was eluted in 20–30 µl RNase-free H_2_O by spinning at maximal speed for 1 min.

Reverse transcription of mature miR-145-5p and snU6 was done according to Biggar et al. with some modifications^54^. In short, 300 ng total RNA was incubated with 5 μL 250 nM stem-loop primer in a total volume of 10 μL. This annealing reaction was carried out at 95 °C for 5 min, followed by 60 °C for 5 min. Samples were immediately centrifuged and held on ice for 1 min. Reverse transcription was done using 1 μL M-MLV Reverse Transcriptase (Invitrogen), 4 μL 5x First Strand Buffer (Invitrogen), 2 μL 100 mM dithiothreitol (Invitrogen) and 1 μL premixed dNTPs (final concentration 25 μM each). Each reaction was brought to a 25 μL total volume using RNase-free water (Qiagen). The following protocol was used for reverse transcription: 16 °C for 30 min, 60 cycles of 20 °C for 30 s, 42 °C for 30 s and 50 °C for 1 s, followed by 85 °C for 5 min, using an Eppendorf Mastercycler. Primer sequences for miRNA qRT-PCR are as follows: miR-145-5p stem-loop primer 5ʹ-CTCACAGTACGTTGGTATCCTTGTGATGTTCGATGCCATATTGTACTGTGAGAGGG

ATTC-3ʹ, miR-145-5p forward 5ʹ- ACACTCCAGCTGGGGTCCAGTTTTCCCAGG-3ʹ, snU6 stem-loop primer 5ʹ- CTCACAGTACGTTGGTATCCTTGTGATGTTCGATGCCATATTGTACTGTGAGAAAA

ATATGGAACGCTT-3ʹ, snU6 forward 5ʹ- ACACTCCAGCTGGGGTGCTCGCTTCGGCAGCACATA-3ʹ, universal reverse 5ʹ- CTCACAGTACGTTGGTATCCTTGTG-3ʹ. Universal reverse primer was used for both miR-145-5p and snU6 amplifications.

Each qRT-PCR reaction contained 12.5 μL 2x SsoFast EvaGreen Supermix (Bio-Rad), 0.8 μL each of 25 μM forward and universal primer, and 4 μL cDNA, filled to 25 μL total with RNase-free water (Qiagen). Samples were amplified using the following protocol: 95 °C for 10 min followed by 40 cycles of 95 °C for 15 s and 60 °C for 1 min, using a Bio-Rad CFX Connect. All samples were run in technical triplicate. Primer validation was done by standard curve efficiency analysis, melt curve analysis, and electrophoresis of qPCR products on a 5% agarose gel to verify product size. Primers for miRNA qRT-PCR were obtained from AlphaDNA. Relative expression analysis was conducted using the CFX Manager^TM^ software or CFX Maestro software using the ΔΔCt method.

For mRNA analysis, total cDNA was constructed using the RT^2^ First Strand Kit (Qiagen) as per the manufacturer’s protocol. Briefly, 2 μL Buffer GE (Qiagen) was used to eliminate genomic DNA from 150 to 200 ng total RNA in RNase-free water in a total volume of 10 μL per sample. Samples were incubated at 42 °C for 5 min and then held on ice for at least 1 min. Reverse transcription was performed by adding 4 μL 5x Buffer BC3 (Qiagen), 1 μL Control P2 (Qiagen), 2 μL RE3 Reverse Transcriptase Mix (Qiagen) and 3 μL RNase-free water (Qiagen), followed by incubation at 42 °C for 15 min and 95 °C for 5 min. Samples were diluted with 91 μL RNase-free water (Qiagen) and stored at -20 °C until use.

For mRNA qRT-PCR, PrimePCR pre-optimized primers (Bio-Rad; sequences proprietary) were used according to the manufacturer’s protocol. Briefly, reactions contained 4 µl total cDNA, 10 µl SsoFast EvaGreen master mix (Bio-Rad), and 1 µl forward/reverse primer mix in a total volume of 20 µl. Amplification was performed at 95 °C for 5 min, followed by 40 cycles of 95 °C for 5 s then 60 °C for 30 s in a Bio-Rad CFX Connect. Relative expression analysis was conducted using the CFX Manager^TM^ software or CFX Maestro software using the ΔΔCt method.

### Western blotting

Protein was isolated by gently homogenizing tissue in 1x RIPA lysis buffer (Sigma) on ice. The lysate was centrifuged at 4 °C at high speed to remove insoluble material. Samples were separated by SDS-PAGE in a 13% gel. Membranes were incubated in 1:1000 CNPase (Abcam), 1:1000 MOG (Abcam), 1:1000 MBP (AbD Serotec) and 1:50,000 alpha-tubulin (Cell Signalling) primary antibodies overnight at 4 °C in Odyssey blocking buffer (Li-Cor Biosciences). Membranes were washed in 1X TBS for 3 ×5 min, then incubated with secondary antibody (IRDye 680RD and 800CW; Li-Cor Biosciences) at 1:20,000 in Odyssey blocking buffer for 1 h at room temperature, and finally washed again in 1X TBS for 3 × 5 min. Membranes were visualized and bands were quantified using the Li-Cor Odyssey CLx Infrared Imaging System.

### Cuprizone

Chronic demyelination of the corpus callosum was achieved in *miR-145*^*+/+*^ and *miR-145*^*−/−*^ animals by using the copper chelating agent cuprizone (oxalic bis-(cyclohexylidenehydrazide)). Cuprizone was milled into normal chow at a final concentration of 0.2% w/w (Envigo) and was fed *ad libitum* starting at P60 to both female and male animals. For acute demyelination, animals were maintained on the cuprizone diet for 6 weeks and were then returned to normal chow for an additional 5 weeks. To achieve chronic demyelination, animals were maintained on the cuprizone diet for 12 weeks, and then returned to normal chow for an additional 5 weeks^23^. During cuprizone treatment, fresh food was provided and old food was removed daily. Control baseline animals were age-matched animals fed with normal chow. Behavioural analyses were performed at P60, P60 + 6 weeks cuprizone, P60 + 6 weeks cuprizone +5 weeks recovery, P60 + 12 weeks cuprizone, and P60 + 12 weeks cuprizone + 5 weeks recovery. Animal weights were recorded and tissues were collected for biochemical, histological and transmission electron microscopy analysis at these same time points.

### Transmission electron microscopy

Mice were anesthetized via intraperitoneal injection of tribromoethanol (Avertin) and perfused transcardially with 5 ml of PBS followed by 10 ml-20 ml of Karnovsky’s fixative (4% paraformaldehyde (PFA), 2% glutaraldehyde and 0.1 M sodium cacodylate in PBS, pH 7.4). Whole brains were extracted and fixed overnight (or until processed) at 4 °C in the same fixative. After fixation, corpus callosum were dissected and cut on either side of the midline under a stereomicroscope into straight segments of 1 mm of length. Specimens were subsequently washed twice in 0.1 M sodium cacodylate buffer for 1 h and once for overnight at room temperature. Segments were post-fixed with 1% osmium tetroxide in 0.1 M sodium cacodylate buffer for 1 h at room temperature, and were then washed in distilled water three times for 5 min. Specimens were dehydrated twice for 20 min for each step in a graded series of ethanol from water through 30%-50%-70%-85%-95% ethanol and twice for 30 minutes in 100% ethanol, followed by twice for 15 min in 50% ethanol/50% acetone and twice for 15 min in 100% acetone. Segments were then infiltrated in 30% Spurr resin/acetone for 20 min and once for 15 h (overnight), then in 50% Spurr resin/acetone for 6 h and in fresh 100% Spurr resin for overnight. Spurr resin was changed twice a day for three days at room temperature. All infiltration steps were performed on a rotator at low speed. Finally, specimens were embedded in fresh liquid Spurr resin and oriented inside the molds and then polymerized overnight at 70 °C. Ultrathin sections (80 nm) were collected onto 200-mesh copper grids and stained with 2% aqueous uranyl acetate and with Reynold’s lead citrate.

### Immunohistochemistry and histology

Mice were anaesthetized and perfused transcardially with 5 ml of PBS followed by 10–20 ml of 4% PFA. Brains were dissected and kept in the same fixative for 48 h and then transferred in 70% ethanol in water. Fixed brains were embedded in paraffin and sectioned by microtome in 20 µm intervals. Whole brain samples were processed at the Louise Pelletier Histology Core Facility, Department of Pathology and Lab Medicine, University of Ottawa, where they were first embedded in paraffin wax with a LOGOS microwave hybrid tissue processor. Paraffin-embedded samples were cut by microtome at a thickness of 20 µm and mounted on slides.

Sections were deparaffinized and rehydrated prior to immunohistochemistry and staining in the following manner: slides were incubated at 59 °C for 30-60 minutes, and were then incubated in in 100% Hemo-D (Histo-Clear, National Diagnostics) for 3 × 5 min, 50% Hemo-D/50% ethanol for 2 × 3 min, 100% ethanol for 2 × 3 min, 95% ethanol for 3 min, 70% ethanol for 3 min, 50% ethanol for 3 min, and then rinsed twice in water. Antigen retrieval was performed for Iba1 immunostaining using Tris-EDTA buffer at pH 9 for 20 min at 95–100 °C using a vegetable steamer. For all antibodies, slides were dried either following antigen retrieval or directly after rehydration, and then rinsed 3 ×5 min in PBS, permeabilized in 0.5% Triton X-100 for 20 min, and then again rinsed 3 × 5 min in PBS. Sections were then blocked for 1 h in blocking solution containing 1% BSA, 10% goat serum, 0.2% Triton X-100 in PBS, followed by primary antibody solution containing 2% BSA, 1% goat serum, 0.2% Triton X-100 and applicable antibodies in PBS at 4 °C overnight. Antibodies were used at the following dilutions: MBP 1:100, GFAP 1:1000, Iba1 1:100. Following primary antibody incubation, sections were washed 3 x with PBS, then incubated for 1 h with AlexaFluor secondary antibodies in the same solution as primary antibody. Sections were then washed once with PBS, counterstained with Dapi at 1:1000 in PBS for 5 min and finally washed 3 × 5 min in PBS. Dako fluorescent mounting medium was applied sparsely directly to the section, and a coverslip was applied.

### Behavioural analyses

Behavioural analyses were performed in cuprizone and baseline animals at the time points indicated above. For all behavioural tests, mice were acclimatized to the testing room for at least 30 min prior to the start of testing.

A Rotarod was used to assess motor coordination. Mice were placed on a rotating, textured rod (IITC Life Science), programmed with the following protocol: starting speed 1 rpm, ramping up to a maximum of 45 rpm over a one minute time span, and then maintaining 45 rpm for an additional minute. Time to falling off the rod (latency to fall) was automatically recorded by magnetic signalling as animals dropped from the rods. Testing was conducted over two consecutive days, with four trials performed per day for a total of 8 trials. Inter-trial intervals were 10 min in duration, when the animals were returned to the home cage. Testing was done in a room lit at 300 lux.

The elevated plus maze (EPM) was used to assess anxiogenic responses. The maze comprised two arms, measuring 6 cm wide and 75 cm long, that crossed perpendicularly in a plus sign shape. One arm is enclosed by 20 cm high walls. The other arm along with the area where the two arms meet were unenclosed platforms. The maze was raised 74 cm off of the floor. Animals were placed singly in the centre of the maze and allowed to explore freely for 10 min while monitored by overhead camera. Movements of each animal were recorded and tracked using Ethovision (Noldus).

### Lentiviral transduction

Primary rat OPCs were plated in OPC medium as above at a density of 7 × 10^3^ cells/well in a 24-well plate or 7 × 10^4^ cells/well in a 6-well plate, and were allowed to recover for 48 h. After 48 h, cells were infected with Lenti-GFP-miR virus (Applied Biological Materials) to overexpress rno-miR-145 or scrambled non-targeting miRNA, or with Lenti-GFP-miR-off virus (Applied Biological Materials) to inhibit rno-miR-145-5p or scrambled non-targeting miRNA inhibitor at a MOI ~ 5 in proliferation medium supplemented with 0.5 µg/mL polybrene (Sigma-Aldrich). After 24 h, cells were washed by performing 3 consecutive 60% media changes with fresh OPC medium. Following an additional 24 h, cells were either fixed or lysed for analysis of OPCs or underwent a 50% media change to 2X differentiation medium for transition to OLs. Differentiating OLs were then fixed or lysed at DD2.5 and DD5 for further analysis.

### Small RNA transfection

MiR-145-5p mimic, scrambled miRNA mimic, miR-145-5p inhibitor, and scrambled miRNA inhibitor were all used at a concentration of 30 nM. MiR-145-5p mimic sequence is identical to mature miR-145-5p based on Sanger miRbase (miRbase.org).

Cells were transfected using INTERFERin siRNA transfection reagent (Polyplus) according to the manufacturer’s protocol. In brief, 30 pmol miRNA mimic or siRNA were prepared in 50 µl Opti-Mem serum-free media (Thermo-Fisher) and vortexed. To this, 1.5 µl INTERFERin was added, the mix was again vortexed, and lipid:RNA complexes were allowed to form for 10 min at room temperature. This transfection mix was added at a ratio of 50 µl for every 1 mL of cell media. For OPCs, transfected cells were assayed 24 h post transfection; for OLs, transfected cells were assayed 5 days post transfection.

### RNA sequencing and gene ontology enrichment analysis

For RNA sequencing (RNAseq), primary rat OPCs were plated and transduced with lentivirus to overexpress miR-145 or scrambled non-targeting miRNA, as described above. RNAseq was performed on *N* = 2 per condition. Quality control and sequencing were performed by StemCore Laboratories, Ottawa Hospital Research Institute. Samples were examined with the Qubit HS RNA Assay (ThermoFisher) and Fragment Analyzer HS NGS assay (Agilent) to determine sample concentration and RNA quality, respectively. Sequencing libraries were constructed using 500 ng RNA from each sample. Library construction was performed using the TruSeq Stranded mRNA Library Prep (Illumina) and sequenced with a NextSeq 500 High Output 75 cycle kit (Illumina).

Bioinformatics analysis of RNAseq raw data was performed by the Ottawa Bioinformatic Core Facility, Ottawa Hospital Research Institute. Reads generated from RNAseq were assigned to transcripts from GENCODE Rnor6.0 using Salmon v0.12.0^55^. Transcript quantifications were then loaded into R using txtimport library for analysis with DESeq2^56^. Following filtration to exclude transcripts with fewer than 5 reads in two or more samples, DESeq2 was used to calculate principal component analysis (PCA), hierarchical clustering, and fold change. PCA was performed using the DESeq2 plotPCA function and rlog-transformed count data. To evaluate hierarchical clustering, a heat map was generated using Pearson correlation between means of CPM normalized expression for each replicate group. Expression differences were calculated using the lfcShrink function^57^. Genes were identified as significantly changed genes using a qvalue (Benjamini-Hochberg corrected *p* value) of <0.05.

Gene ontology (GO) biological process functional enrichment analysis of the significantly changed genes was performed using g:Profiler^58^. Genes were separated into two sets defined as significantly upregulated and significantly downregulated with a fold change of 1.5 or greater, and were assessed by ordered query weighted by significance. GO terms used to identify targets for validation by qRT-PCR were significantly enriched and identified based on known relevance to OL biology.

### Imaging and quantification

Fluorescence images were taken using an Axio Imager M1 microscope with an AxioCamHR HRm Rev.2 camera and Axiovision 4.8.2 software. Confocal images were obtained using a Zeiss LSM 510 Meta DuoScan microscope and Zen 8.0 software. All electron micrographs of the corpus callosum taken with a transmission electron microscope (Hitachi 7100) at ×4000 and ×10,000 magnifications. Both immunohistochemistry and histology tissue samples were imaged using a Zeiss AxioScan slide scanner with a Colibri 7 camera and Zen 2.6 slidescan software. All images were analysed using ImageJ, and treatment conditions were blinded for all imaging analyses.

For primary culture, two coverslips were analysed for each n. For images taken at ×20, 20–25 images were quantified per n; for those taken at ×10, 5–10 images were quantified per n. Sholl analysis was used to quantify branching complexity by overlaying concentric circles at equal increments from the cell body, and branch intersections were counted at each ring for 30 cells per n. Total membrane area was measured by tracing MBP^+^ cell outline and then calculating area for 20–30 cells per n.

From TEM, G-ratios were calculated by measuring axon and axon + myelin areas, converting to diameter, and dividing axon diameter/axon + myelin diameter. For heavily myelinated samples, ~300 axons were measured per n; for sparsely myelinated samples, all myelinated axons were measured in at least 90 images taken at 10 000x. For myelinated axons/area, total myelinated axons were counted in ~30-60 images taken at 10 000x.

For immunohistochemistry, whole brains were sectioned coronally from the corpus callosum genu to the rostral areas of the corpus callosum adjacent the thalamus and hypothalamus. GFAP+ and Iba1+ cells were quantified in the corpus callosum (including both medial and lateral areas), and the outline of the corpus callosum was traced to determine its size. The number of GFAP+ or Iba+ cells was then calculated per 1000 µm2 total corpus callosum for 4 sections per animal.

### Statistics and reproducibility

Statistical analyses were performed using Prism 6 GraphPad software except for qRT-PCR analyses, which was calculated as indicated above. Pair-wise comparisons were done by two-tailed Student’s *t* test using *n* = 3–6, as indicated. Comparisons for >2 conditions were done using one-way or two-way analysis of variance (ANOVA) followed by appropriate post hoc testing using *n* = 3–6, as indicated. Linear regression analyses were conducted for g-ratio assessments. Kaplan-Meier curves were analysed using the Mantel-Cox test. Error on mean values represent ± SEM, unless otherwise indicated. *P* values of <0.05 were considered significant; ns = not significant, asterisks delineate significance values as follows: *p < 0.05, **p < 0.01, ***p < 0.001, ****p < 0.0001.

### Reporting summary

Further information on research design is available in the Nature Portfolio Reporting Summary linked to this article.

### Supplementary information

Supplementary Information

Description of Additional Supplementary Files

Supplementary Data 1

Supplementary Data 2

Supplementary Data 3

Reporting Summary

## Data Availability

The data that support the findings of this study are available from the corresponding author upon request. RNAseq gene expression data have been deposited and approved by GEO, with accession number GSE268808 and data at the following link: https://www.ncbi.nlm.nih.gov/geo/query/acc.cgi?acc=GSE268808. Uncropped and unedited blot/gel images used or presented in Supplementary Fig. 2 have been included as Supplementary Fig. 7.
