# Supplementary material for: Loss of miR-145 promotes remyelination and functional recovery in a model of chronic central demyelination
Source: Commun Biol. 2024 Jul 4;7:813. doi: 10.1038/s42003-024-06513-x (PMC11224363; doi:10.1038/s42003-024-06513-x)
Supplement: Supplementary file 1 — Supplementary Information [file 42003_2024_6513_MOESM1_ESM.pdf]

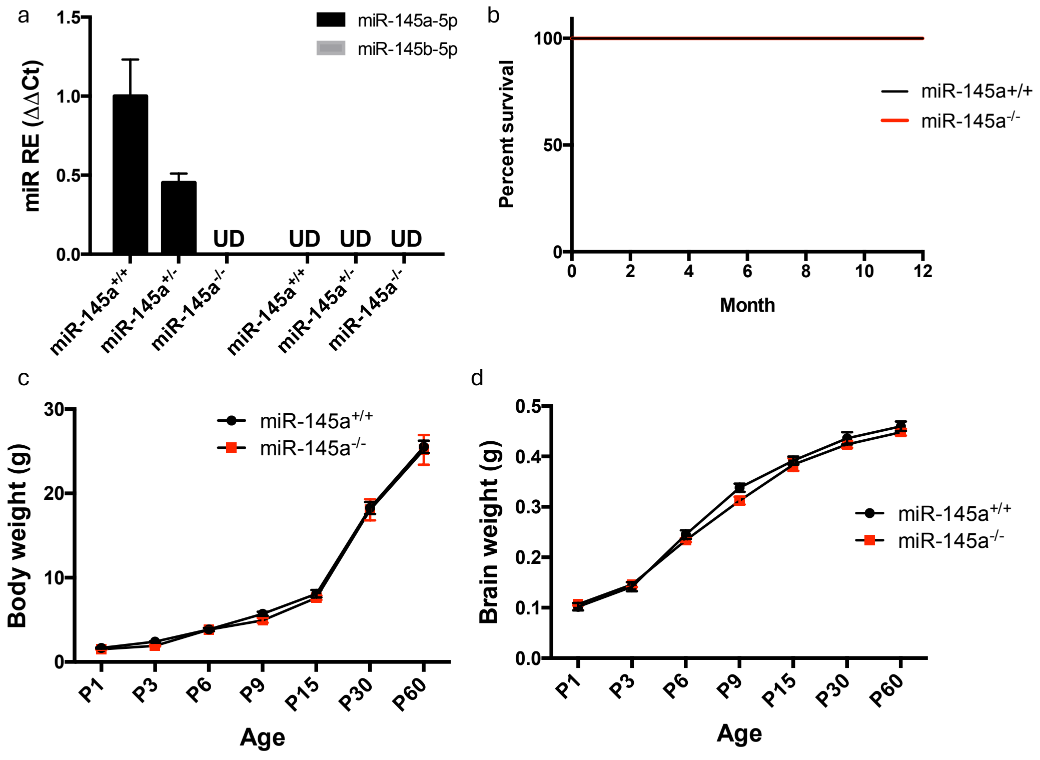

Supplementary Figure 1. *miR-145$^{-/-}$* mice exhibit normal survival and growth. a. Relative expression analysis of miR-145-5p and miR-145b-5p in *miR-145$^{+/+}$*, *miR-145$^{+/-}$* and *miR-145$^{-/-}$* CNS tissue by qRT-PCR. Analysed by ΔΔCt, normalized to snU6. N=3. UD = undetectable. b. Kaplan-Meier survival curve for *miR-145$^{+/+}$* and *miR-145$^{-/-}$* mice. N=10, Mantel-Cox test. c/d. Body weights (c) and whole brain weights (d) of *miR-145$^{+/+}$* and *miR-145$^{-/-}$* mice from neonate to adult. No significant differences based on multiple t-tests of two-way ANOVA using Holm-Sidak method. All values represent mean ± SEM. c. N=5-10. d. N=5-7.

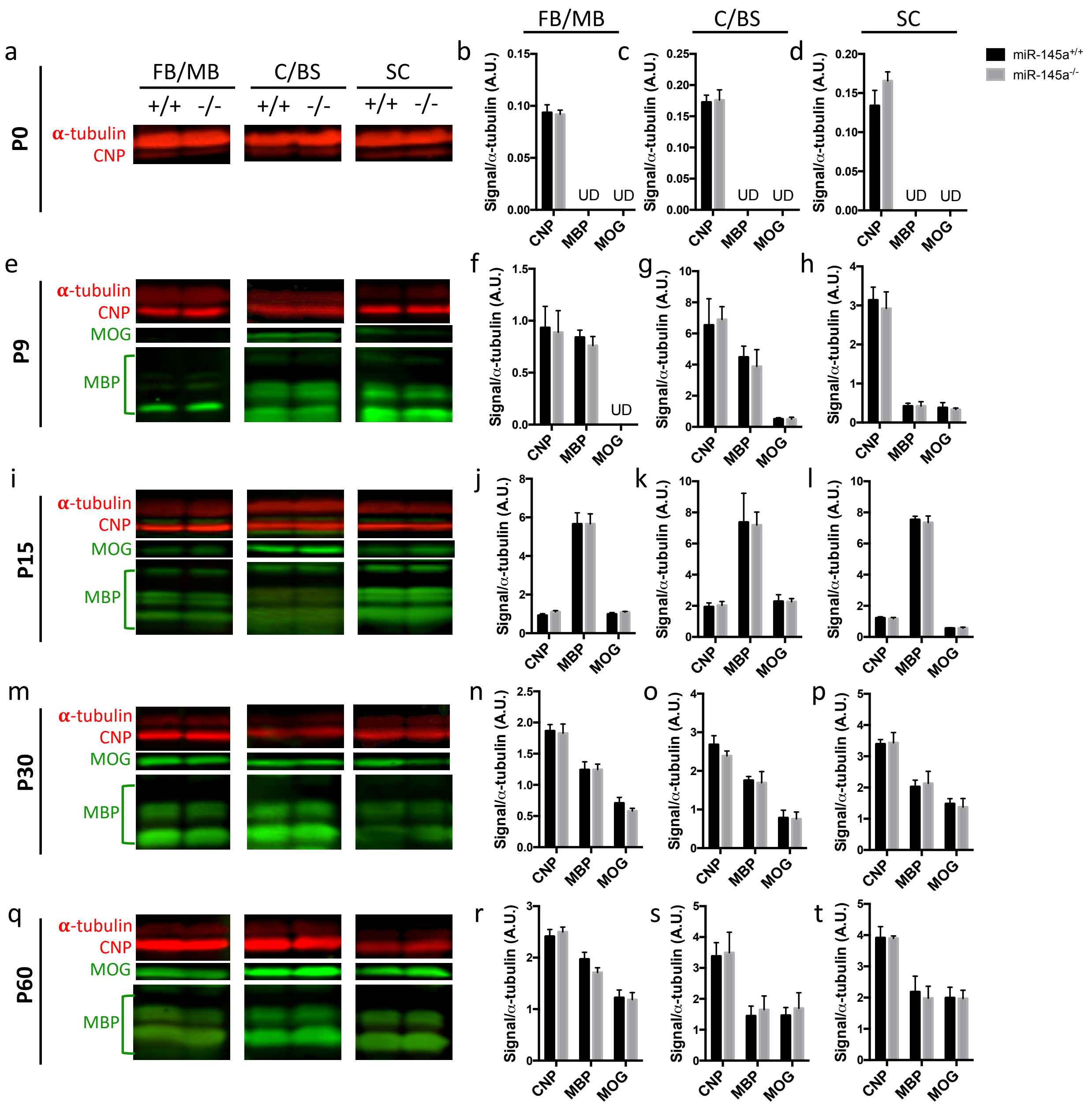

Supplementary Figure 2. Changes in CNS myelin protein expression were not detected with loss of mir-145 during development. a-t. Representative western blots (a, e, i, m, q) and quantifications (b-d, f-h, j-l, n-p, r-t) for intermediate myelin marker CNP and late myelin markers MBP and MOG in forebrain and midbrain (FB/MB), cerebellum and brainstem (C/BS) and spinal cord (SC) from *miR-145*$^{+/+}$ and *miR-145*$^{-/-}$ neonate (P0), juvenile (P9, P15), early adult (P30) and mature adult (P60) animals. N=5, Student's t-test. All data represent mean ± SEM.

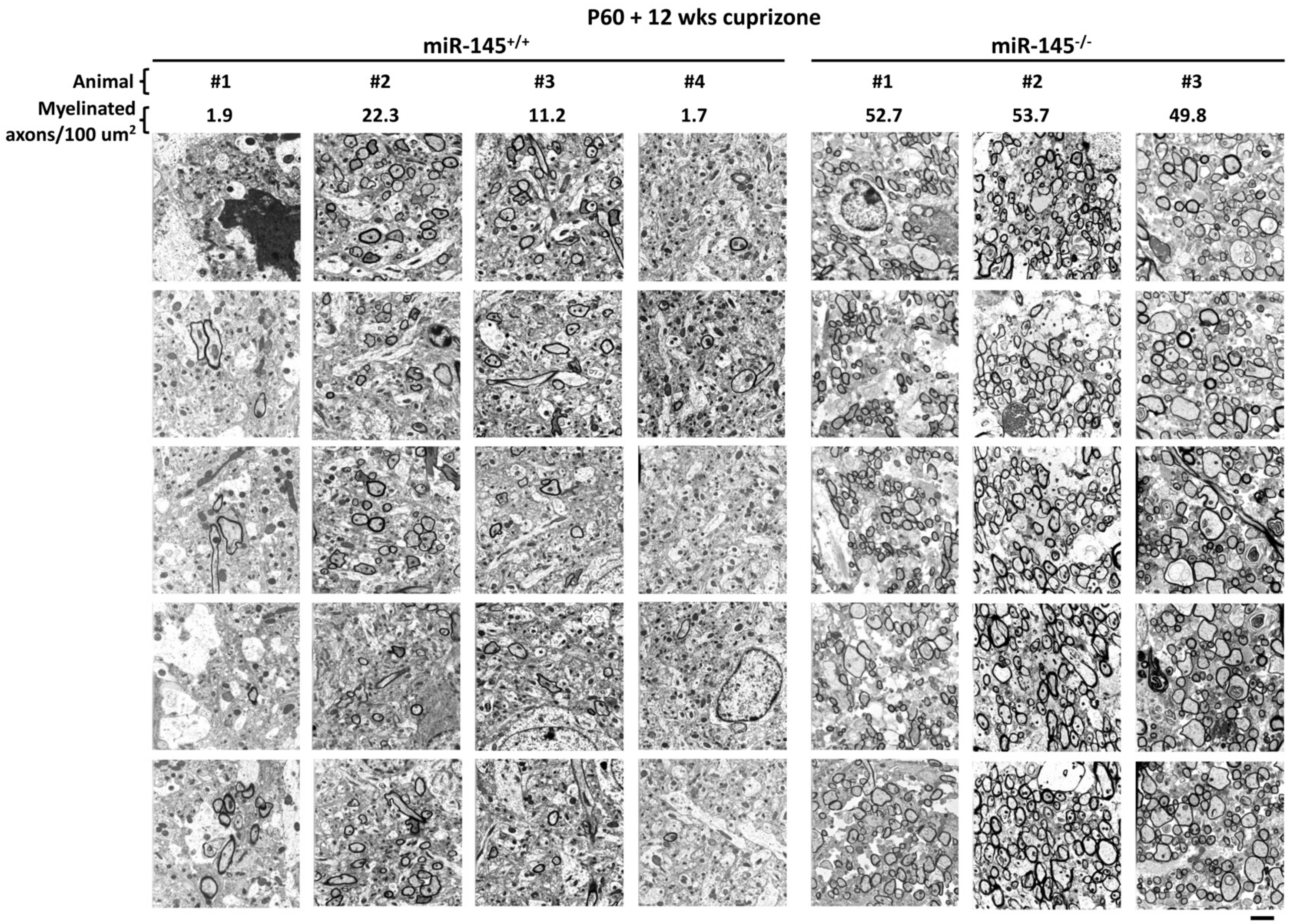

Supplementary Figure 3. Transmission electron microscope micrographs of corpus callosum after 12 weeks chronic cuprizone exposure in *miR-145$^{+/+}$* and *miR-145$^{-/-}$* animals. Individual animals are represented by number (#1, #2, etc.) with corresponding total myelinated axons quantified per 100 um^2 (see also Supplementary Table 1).

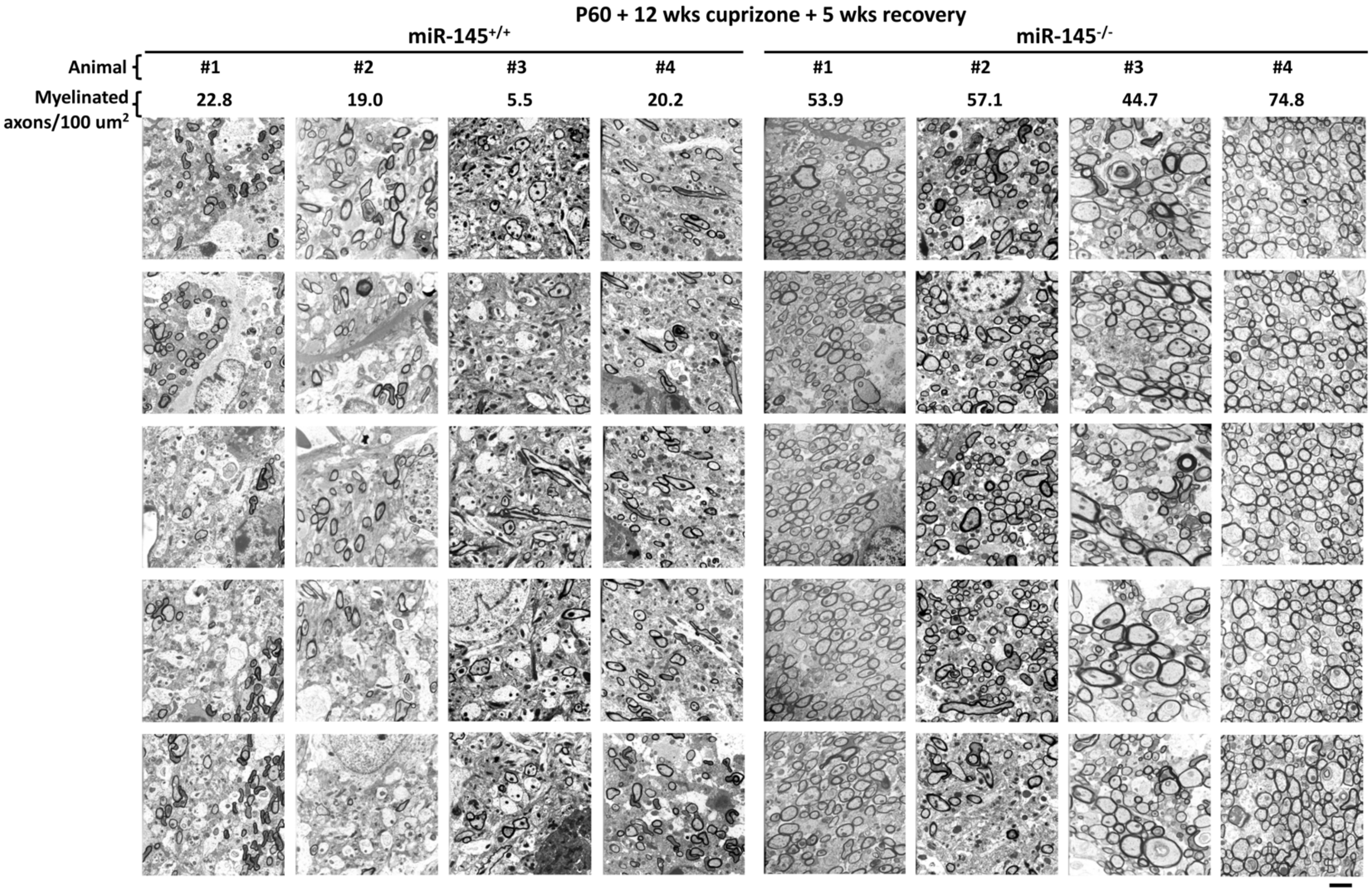

Supplementary Figure 4. Transmission electron microscope micrographs of corpus callosum after 12 weeks chronic cuprizone exposure followed by 5 weeks recovery in *miR-145*$^{+/+}$ and *miR-145*$^{-/-}$ animals. Individual animals are represented by number (#1, #2, etc.) with corresponding total myelinated axons quantified per 100 um^2 (see also Supplementary Table 1).

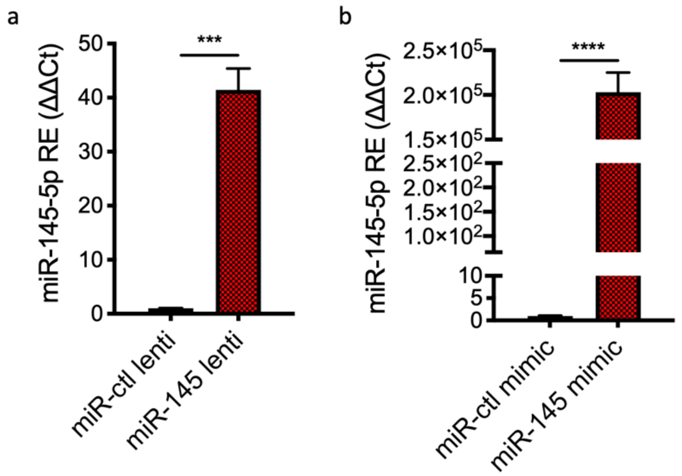

Supplementary Figure 5. Validation of miR-145 mimic. a. MiR-145-5p relative expression in lentivirus-transduced OLs on differentiation day 2.5 (DD2.5)**.** b. MiR-145-5p relative expression in OLs transfected with 30 nM miRNA mimic (miR-ctl) or miR-145 mimic on DD5. a-b. Analysis by qRT-PCR using ΔΔCt method, normalized to snU6. N=4, ***=p<0.001, ****=p<0.0001, Student’s t-test.

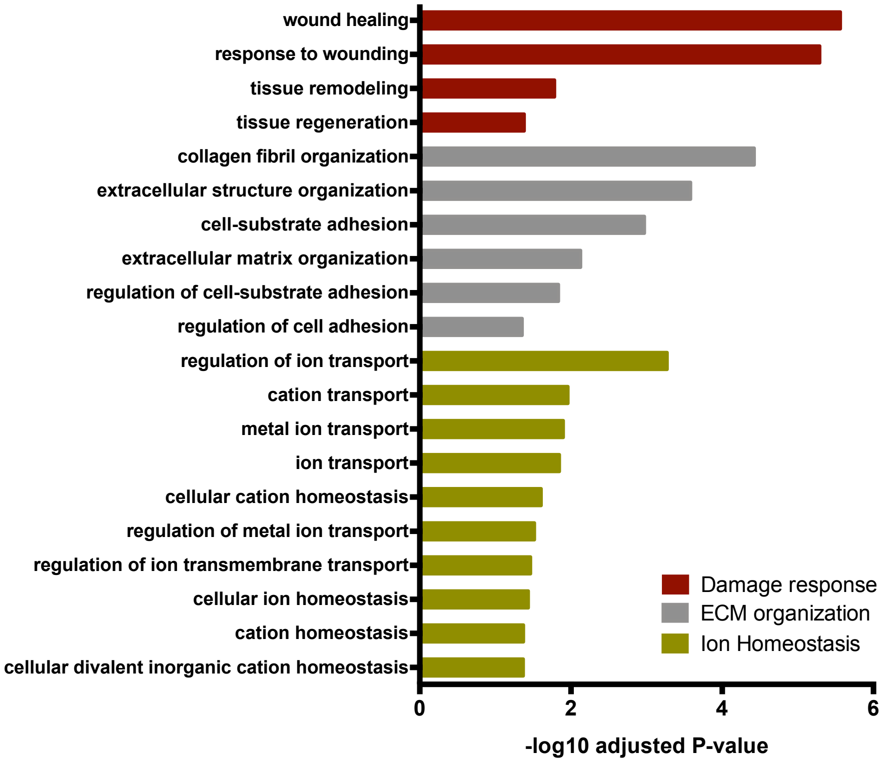

Supplementary Figure 6. Significantly enriched GO terms for genes upregulated in miR-145 OLs. Genes differentially upregulated in miR-145 OLs relative to miR-ctl OLs were assessed for enriched gene ontology terms.

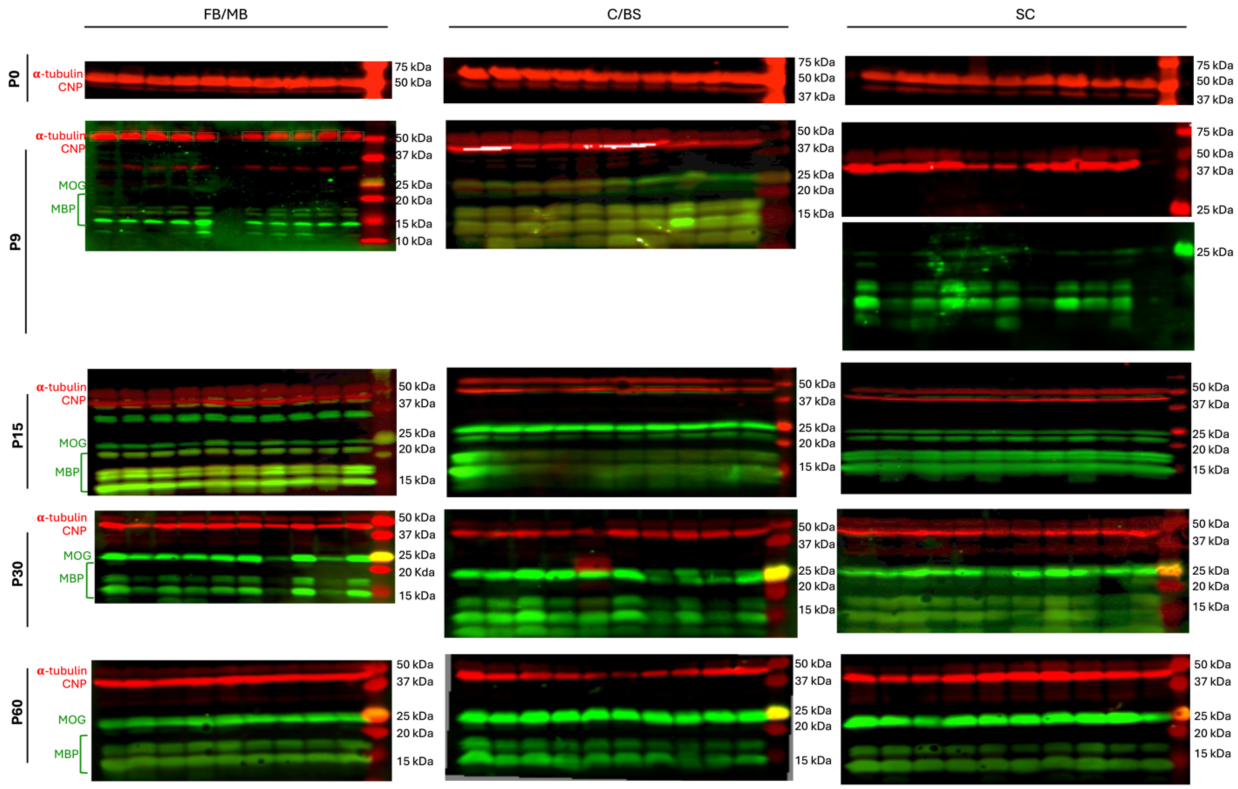

Supplementary Figure 7. Full Western blots for myelin proteins quantified in Supplementary Figure 2. Western blots for intermediate myelin marker CNP and later myelin markers MBP and MOG in in forebrain and midbrain (FB/MB), cerebellum and brainstem (C/BS) and spinal cord (SC) from *miR-145*$^{+/+}$ and *miR-145*$^{-/-}$ neonate (P0), juvenile (P9, P15), early adult (P30) and mature adult (P60) animals. Lanes alternate genotype (i.e. lane 1 = *miR-145*$^{+/+}$, lane 2 = *miR-145*$^{-/-}$, lane 3 = *miR-145*$^{+/+}$, lane 4 = *miR-145*$^{-/-}$, etc.). Note that a dual channel image was not available for P9 spinal cord (SC); therefore, individual channels of the same blot are shown.
