# Supplementary material for: Loss of miR-145 promotes remyelination and functional recovery in a model of chronic central demyelination
Source: Commun Biol. 2024 Jul 4;7:813. doi: 10.1038/s42003-024-06513-x (PMC11224363; doi:10.1038/s42003-024-06513-x)
Supplement: Supplementary file 2 — Description of Additional Supplementary Files [file 42003_2024_6513_MOESM2_ESM.pdf]

Description of Additional Supplementary Files

File: Supplementary Data 1.

Description: Quantification of myelinated axons/100 um2 per animal in normal and cuprizone-treated animals.

File: Supplemental Data 2.

Description: All significantly enriched gene ontology terms for genes downregulated with miR-145-5p overexpression in OLs.

File: Supplemental Data 3.

Description: All significantly enriched gene ontology terms for genes upregulated with miR-145-5p overexpression in OLs.
