# Supplementary material for: Loss of miR-145 promotes remyelination and functional recovery in a model of chronic central demyelination
Source: Commun Biol. 2024 Jul 4;7:813. doi: 10.1038/s42003-024-06513-x (PMC11224363; doi:10.1038/s42003-024-06513-x)
Supplement: Supplementary file 6 — Reporting Summary [file 42003_2024_6513_MOESM6_ESM.pdf]

nature portfolio

Corresponding author(s): Rashmi Kothary

Last updated by author(s): Jun 3, 2024

Reporting Summary

Nature Portfolio wishes to improve the reproducibility of the work that we publish. This form provides structure for consistency and transparency in reporting. For further information on Nature Portfolio policies, see our Editorial Policies and the Editorial Policy Checklist.

Statistics

For all statistical analyses, confirm that the following items are present in the figure legend, table legend, main text, or Methods section.

n/a	Confirmed	
☐	☒	The exact sample size (*n*) for each experimental group/condition, given as a discrete number and unit of measurement
☐	☒	A statement on whether measurements were taken from distinct samples or whether the same sample was measured repeatedly
☐	☒	The statistical test(s) used AND whether they are one- or two-sided<br>*Only common tests should be described solely by name; describe more complex techniques in the Methods section.*
☐	☒	A description of all covariates tested
☐	☒	A description of any assumptions or corrections, such as tests of normality and adjustment for multiple comparisons
☐	☒	A full description of the statistical parameters including central tendency (e.g. means) or other basic estimates (e.g. regression coefficient) AND variation (e.g. standard deviation) or associated estimates of uncertainty (e.g. confidence intervals)
☐	☒	For null hypothesis testing, the test statistic (e.g. *F*, *t*, *r*) with confidence intervals, effect sizes, degrees of freedom and *P* value noted<br>*Give P values as exact values whenever suitable.*
☒	☐	For Bayesian analysis, information on the choice of priors and Markov chain Monte Carlo settings
☒	☐	For hierarchical and complex designs, identification of the appropriate level for tests and full reporting of outcomes
☐	☒	Estimates of effect sizes (e.g. Cohen's *d*, Pearson's *r*), indicating how they were calculated

Our web collection on statistics for biologists contains articles on many of the points above.

Software and code

Policy information about availability of computer code

Data collection	For imaging, data were generated using ImageJ, as indicated in the manuscript.
Data analysis	For all analyses except qRT-PCr and RNAseq, GraphPad Prism 6 software was used for statistical analysis, as described in the manuscript. For qRT-PCR, Bio-Rad software CFX Manager or CFX Maestro were used, as indicated in the manuscript. For RNAseq, and as indicated in the manuscript, the following were used: GENCODE Rnor6.0, Salmon v0.12.0, DESeq2, and g:Profiler.

For manuscripts utilizing custom algorithms or software that are central to the research but not yet described in published literature, software must be made available to editors and reviewers. We strongly encourage code deposition in a community repository (e.g. GitHub). See the Nature Portfolio guidelines for submitting code & software for further information.

Data

Policy information about availability of data

All manuscripts must include a data availability statement. This statement should provide the following information, where applicable:

- Accession codes, unique identifiers, or web links for publicly available datasets
- A description of any restrictions on data availability
- For clinical datasets or third party data, please ensure that the statement adheres to our policy

The data that support the findings of this study are available from the corresponding author upon request. RNAseq gene expression data have been deposited and approved by GEO, with accession number GSE268808 and data at the following link:

https://www.ncbi.nlm.nih.gov/geo/query/acc.cgi?acc=GSE268808

Human research participants

Policy information about studies involving human research participants and Sex and Gender in Research.

Reporting on sex and gender	Lesion tissue from humans suffering from SPMS and RRMS were used - both male and female were included.
Population characteristics	See Table 1 of the manuscript.
Recruitment	N/A
Ethics oversight	Tissue samples were collected from healthy donors and MS patients with full ethical approval (BH07.001) and informed consent from the Centre de recherche du Centre hospitalier de l'Université de Montréal ethics committee.

Note that full information on the approval of the study protocol must also be provided in the manuscript.

Field-specific reporting

Please select the one below that is the best fit for your research. If you are not sure, read the appropriate sections before making your selection.

☒ Life sciences ☐ Behavioural & social sciences ☐ Ecological, evolutionary & environmental sciences

For a reference copy of the document with all sections, see nature.com/documents/nr-reporting-summary-flat.pdf

Life sciences study design

All studies must disclose on these points even when the disclosure is negative.

Sample size	Sample sizes were not statistically predetermined but all sample sizes meet or exceed those found in previously published literature, by method.
Data exclusions	No data were excluded.
Replication	Multiple Ns were assessed for all experiments, where each N represents a biological replicate. For human experiments, N = a demyelinated lesion or an area of white matter; for mouse tissue and demyelination experiments, N = one individual mouse; for primary cell culture, N = enriched OPC/OL cultures from one individual mouse; for immortalized cell culture; N = cells from separate passages. For mouse and primary cell culture experiments, animals were used from multiple breeding pairs and across multiple litters. All findings were replicated successfully.
Randomization	Both individual animals and primary cell populations were randomly assigned to experimental groups.
Blinding	Investigators were blinded to experimental conditions for all imaging analyses, as indicated in the manuscript.

Reporting for specific materials, systems and methods

We require information from authors about some types of materials, experimental systems and methods used in many studies. Here, indicate whether each material, system or method listed is relevant to your study. If you are not sure if a list item applies to your research, read the appropriate section before selecting a response.

Materials & experimental systems

n/a	Involved in the study	
☐	☒	Antibodies
☐	☒	Eukaryotic cell lines
☒	☐	Palaeontology and archaeology
☐	☒	Animals and other organisms
☒	☐	Clinical data
☒	☐	Dual use research of concern

Methods

n/a	Involved in the study	
☒	☐	ChIP-seq
☒	☐	Flow cytometry
☒	☐	MRI-based neuroimaging

Antibodies

Antibodies used	MBP (clone 12, AbD Serotec, catalog # MCA-409S), CNPase (clone 11-5B, abcam, catalog # ab6319) , MOG (abcam, catalog #

Antibodies used	ab32760), alpha-Tubulin (clone DM1A, Cell Signaling, catalog # mAb 3873), Olig2 (EMD Millipore, catalog # ab9610), cleaved-caspase 3 (clone 5A1E, Cell Signaling, catalog # mAb 9664), GFAP (abcam, catalog # ab7260), Iba1 (abcam, catalog # ab153696)
Validation	All antibodies used were validated by the manufacturer for the applications use in our study, and have been similarly used in previous publications by us and others.

Eukaryotic cell lines

Policy information about cell lines and Sex and Gender in Research

Cell line source(s)	Oli-neu cells were generated by Jung et al (1995), as referenced in the manuscript.
Authentication	The cell line was not authenticated.
Mycoplasma contamination	The line was deemed free of mycoplasma contamination using a commerically available PCR detection kit.
Commonly misidentified lines (See ICLAC register)	N/A

Animals and other research organisms

Policy information about studies involving animals; ARRIVE guidelines recommended for reporting animal research, and Sex and Gender in Research

Laboratory animals	See "Animals" and "Cell cluture" sections in methods of manuscript.
Wild animals	N/A
Reporting on sex	Animals of both sex were included in our study.
Field-collected samples	N/A
Ethics oversight	The University of Ottawa Animal Care Committee approved all experimental protocols involving animals. The protocols conformed to or exceeded those defined in the Canadian Council on Animal Care's Guide to the Care and Use of Experimental Animals, and the Animals for Research Act.

Note that full information on the approval of the study protocol must also be provided in the manuscript.
